# Inhibition of the hERG potassium channel by phenanthrene: a polycyclic aromatic hydrocarbon pollutant

**DOI:** 10.1007/s00018-021-03967-8

**Published:** 2021-11-02

**Authors:** Ehab Al-Moubarak, Holly A. Shiels, Yihong Zhang, Chunyun Du, Oliver Hanington, Stephen C. Harmer, Christopher E. Dempsey, Jules C. Hancox

**Affiliations:** 1 School of Physiology, Pharmacology and Neuroscience, University of Bristol, Biomedical Sciences Building, University Walk, Bristol, BS8 1TD UK; 2 Division of Cardiovascular Sciences, University of Manchester, Manchester, UK; 3 School of Biochemistry, University of Bristol, Bristol, UK

**Keywords:** Hydrocarbon, *KCNH2*, PAH, Phenanthrene, Pollutant, Potassium channel

## Abstract

The lipophilic polycyclic aromatic hydrocarbon (PAH) phenanthrene is relatively abundant in polluted air and water and can access and accumulate in human tissue. Phenanthrene has been reported to interact with cardiac ion channels in several fish species. This study was undertaken to investigate the ability of phenanthrene to interact with *hERG* (*human Ether-à-go-go-Related Gene*) encoded Kv11.1 K^+^ channels, which play a central role in human ventricular repolarization. Pharmacological inhibition of hERG can be proarrhythmic. Whole-cell patch clamp recordings of hERG current (I_hERG_) were made from HEK293 cells expressing wild-type (WT) and mutant hERG channels. WT I_hERG1a_ was inhibited by phenanthrene with an IC_50_ of 17.6 ± 1.7 µM, whilst I_hERG1a/1b_ exhibited an IC_50_ of 1.8 ± 0.3 µM. WT I_hERG_ block showed marked voltage and time dependence, indicative of dependence of inhibition on channel gating. The inhibitory effect of phenanthrene was markedly impaired by the attenuated inactivation N588K mutation. Remarkably, mutations of S6 domain aromatic amino acids (Y652, F656) in the canonical drug binding site did not impair the inhibitory action of phenanthrene; the Y652A mutation augmented I_hERG_ block. In contrast, the F557L (S5) and M651A (S6) mutations impaired the ability of phenanthrene to inhibit I_hERG_, as did the S624A mutation below the selectivity filter region. Computational docking using a cryo-EM derived hERG structure supported the mutagenesis data. Thus, phenanthrene acts as an inhibitor of the hERG K^+^ channel by directly interacting with the channel, binding to a distinct site in the channel pore domain.

**Supplementary Information:**

The online version contains supplementary material available at 10.1007/s00018-021-03967-8.

## Introduction

Humanity’s dependence on fossil fuels has led to the near-ubiquitous pollution of the environment with petrochemicals. Exposure to polluted air is associated with a range of adverse cardiovascular events including heart attacks, strokes and irregular heart rhythms, particularly in people already at risk for these conditions [1]. The mechanisms of cardiotoxicity are complex and can be attributed in part, to nanoparticles containing polycyclic aromatic hydrocarbons (PAHs) that are produced during combustion of coal, petroleum, diesel, tobacco and by cooking food [1]. The adverse health effects of PAHs have long been recognized, but with a focus primarily on carcinogenic compounds, such as benzo(a)pyrene (BAP) [2] and other large molecular weight members (i.e. 4+ rings) of the PAH family. In contrast, the noncarcinogenic smaller PAHs have largely been ignored by the human biomedicine community. These smaller 2- and 3-ring compounds are several orders of magnitude more abundant than the larger 4-ring + PAHs in the fossil fuel sources that feed urban air pollution [3], but are not routinely measured in air pollution; when they are, their ubiquity and abundance are clear. Other than 2-ringed naphthalenes, phenanthrene with 3-rings and its alkylated homologs make up the largest fraction of the measured PAHs in virtually all fuel-related sources. This includes fine particulate matter (e.g. PM_2.5_), such as diesel exhaust particles and broadly collected urban PM_2.5_ (e.g. [4, 5]). More importantly, phenanthrenes are volatile so are even more abundant in the vapor phase of vehicle exhaust and urban air (e.g. [6, 7]).

Although the phenanthrenes (and other smaller PAHs) have not been considered fully in the etiology of air pollution-related cardiovascular disease in humans, for nearly three decades they have been a major focus of the cardiotoxicity syndrome that occurs in developing fish following aquatic oil spills (reviewed in Ref. [8]). Oil spills, such as the 1989 Exxon Valdez tanker grounding and the 2010 Deepwater Horizon wellhead blowout released large quantities of PAHs directly into the marine environment, bringing global attention to the cardiotoxic effects of PAHs for fish [1, 9, 10]. Exposure of zebrafish embryos to 3-ringed PAHs was found to produce bradycardia and arrhythmia consistent with atrioventricular block; the authors suggested *Ether-à-go-go-Related Gene* (ERG) encoded K^+^ channels as a potential target that might mediate such effects [9]. Crude oil extracts from the Deepwater Horizon disaster were then shown to exert direct effects on the function of tuna cardiac myocytes: they produced impairment of calcium cycling and action potential prolongation, associated with inhibition of the rapid delayed rectifier K^+^ current (I_Kr_, the ion current carried by *erg* [11]). Subsequent work isolated the 3-ringed PAH phenanthrene as the key moiety involved in these effects producing a concentration-dependent ventricular action potential prolongation and inhibition of I_Kr_ [12]. Phenanthrene has subsequently been shown to increase sodium current and reduce both calcium current and I_Kr_ in ventricular myocytes from rainbow trout [13], with ventricular action potential prolongation and reductions in calcium current and I_Kr_ with phenanthrene also recently reported for brown trout cardiomyocytes [14]. Furthermore, phenanthrene has recently been reported to inhibit calcium current and I_Kr_ in zebrafish cardiomyocytes [15]. There is debate as to whether toxic effects of pollutants on fish from crude oil may largely result from membrane disruption rather than interaction with specific receptors (e.g. [16]). Whilst the effects of phenanthrene on specific cardiac ion channel currents may be consistent with a direct rather than nonselective effect, definitive evidence for this is currently lacking.

**Fig. 1:**
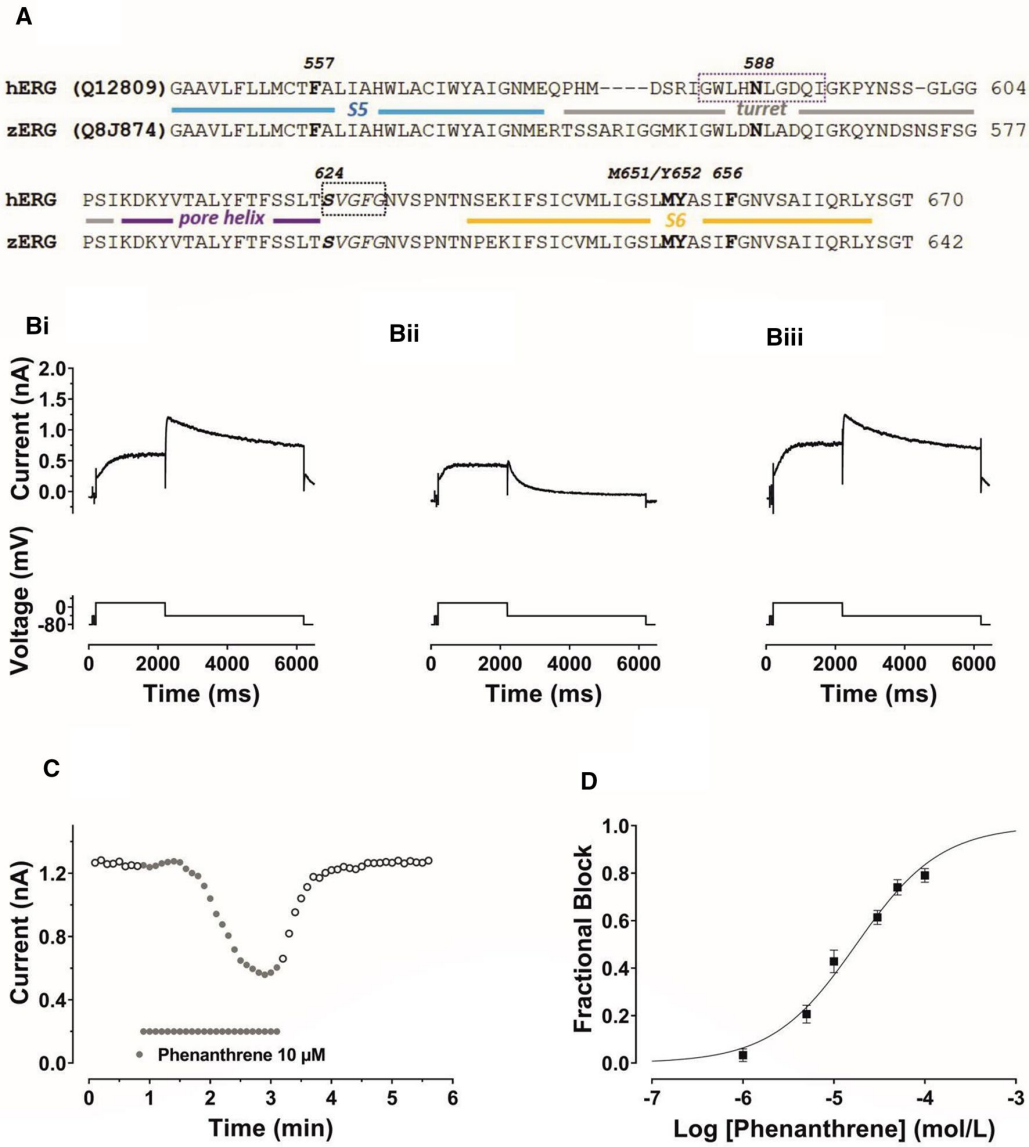
**A** Sequence alignments of zERG and hERG showing very high homology in the pore helix, S5 and S6 regions that contribute to the canonical drug binding site and residues investigated here for phenanthrene. The structural elements of the channel pore domain are coloured to match the structural figure in Fig. 6a. **B** Upper traces show **Bi** Control I_hERG_, **Bii** I_hERG_ during application of 10 µM phenanthrene (Phen), **Biii** Current during washout of Phen. I_hERG_ was elicited by a voltage protocol shown as lower traces, comprised of a 2 s depolarizing pulse to +20 mV, followed by repolarization to −40 mV. **C** Time course of I_hERG_ inhibition: plots of I_hERG_ tails in control, during phenanthrene application and washout. **D** Concentration response relation for phenanthrene inhibition of I_hERG_. Six different concentrations between 1 µM and 100 µM were tested (1 µM, 5 µM, 10 µM, 30 µM, 50 µM and 100 µM). At least 5 replicates per concentration were obtained. The data were fitted with Eq. 2. Phenanthrene inhibited I_hERG_ carried by the hERG1a isoform with a half-maximal inhibitory concentration (IC_50_) of 17.6 ± 1.7 µM and Hill coefficient of 0.94 ± 0.09

The reported effects of phenanthrene on fish I_Kr_ are of relevance to human cardiovascular health because I_Kr_ is known to be of key importance not only in animal model species, but also to normal human ventricular repolarization [17, 18]. I_Kr_ channels are comprised of tetramers of pore-forming Kv11.1 subunits encoded by *hERG* (*human Ether-à-go-go-Related Gene*, alternative nomenclature *KCNH2*; [19, 20]). Loss- and gain-of function mutations to *hERG*, respectively underlie forms of inherited long and short QT syndromes (LQTS and SQTS, respectively) [19, 21]. Moreover, due to unique structural features, hERG channels are highly susceptible to pharmacological blockade, leading to a drug-induced form of LQTS, with its associated risk of dangerous ventricular arrhythmias [17, 18]. Phenanthrene is relatively abundant in polluted air and water. It is lipophilic and can access and accumulate in human tissue via skin and mucus membranes [1]. Given the known susceptibility of hERG to pharmacological blockade and the fact that fish I_Kr_ is inhibited by phenanthrene, it is reasonable to expect that hERG itself may be susceptible to phenanthrene inhibition. Furthermore, alignment of fish ERG and hERG sequences reveals a high level of identity in regions of the channel that constitute the canonical drug binding site (Fig. 1A and [1]). It therefore seems likely that human I_Kr_/hERG also may be susceptible to inhibition by phenanthrene. Accordingly, this study was undertaken: (i) to determine the propensity or otherwise of phenanthrene to inhibit ionic current (I_hERG_) carried by recombinant hERG channels; (ii) to explore the underlying mechanism of any observed effect. Some of this work has been published in meeting abstract form [22, 23].

## Materials and methods

### Maintenance of mammalian cell lines and cell transfection

As in previous studies (e.g. [24–26]), experiments were performed on HEK293 cells stably expressing WT hERG [27] or transiently transfected with hERG mutant cDNAs. The hERG stable line was generously provided by Professor Craig January [27]. HEK293 cells (ECACC, Porton Down, UK) were transiently transfected with cDNA plasmids using Lipofectamine 2000 (Invitrogen, Paisley, UK) according to the manufacturer’s instructions. Expression plasmid encoding CD8 was also added (in pIRES, donated by Dr I Baró, University of Nantes, France) as a marker for successful transfection. Cells were passaged using enzyme-free cell dissociation solution (Millipore, Watford, UK) and plated onto sterilized shards of glass coverslips in 40-mm petri dishes containing a modification of Dulbecco minimum essential medium with Glutamax-1 (DMEM; Invitrogen, Paisley, UK). This was supplemented with 10% fetal bovine serum (Gibco, Gloucester, UK), 50 μg/mL gentamycin (Invitrogen, Paisley, UK) and 400 μg/mL geneticin (G418, Invitrogen, Paisley, UK) for the WT hERG-expressing line. For experiments utilizing cells transiently transfected with hERG mutants, recordings were performed 24-72 h after transfection. Successfully transfected cells (positive to CD8) were identified using Dynabeads^®^ (Invitrogen, Paisley, UK) [25, 26]. The N588K, S624A, Y652A, M651A and F557L mutations to hERG1a have all been used in prior studies from our laboratory (e.g. [24–26, 28, 29]), as has hERG1a/1b co-expression [25, 30]. Zebrafish ERG (zERG) was synthesised and provided in pcDNA3.1 by Genscript (Leiden, The Netherlands; NCBI Reference Sequence: NM_212837.1).

The F656V mutation was made to hERG1a as described in Ref. [31]. F656T was made using QuikChange (Agilent) mutagenesis using conditions described in Ref. [31] and the following primer sequences:

forward-5′-GTATGCTAGCATCACCGGCAACGTGTCG-3′

reverse-5′-CGACACGTTGCCGGTGATGCTAGCATAC-3′

**Fig. 2:**
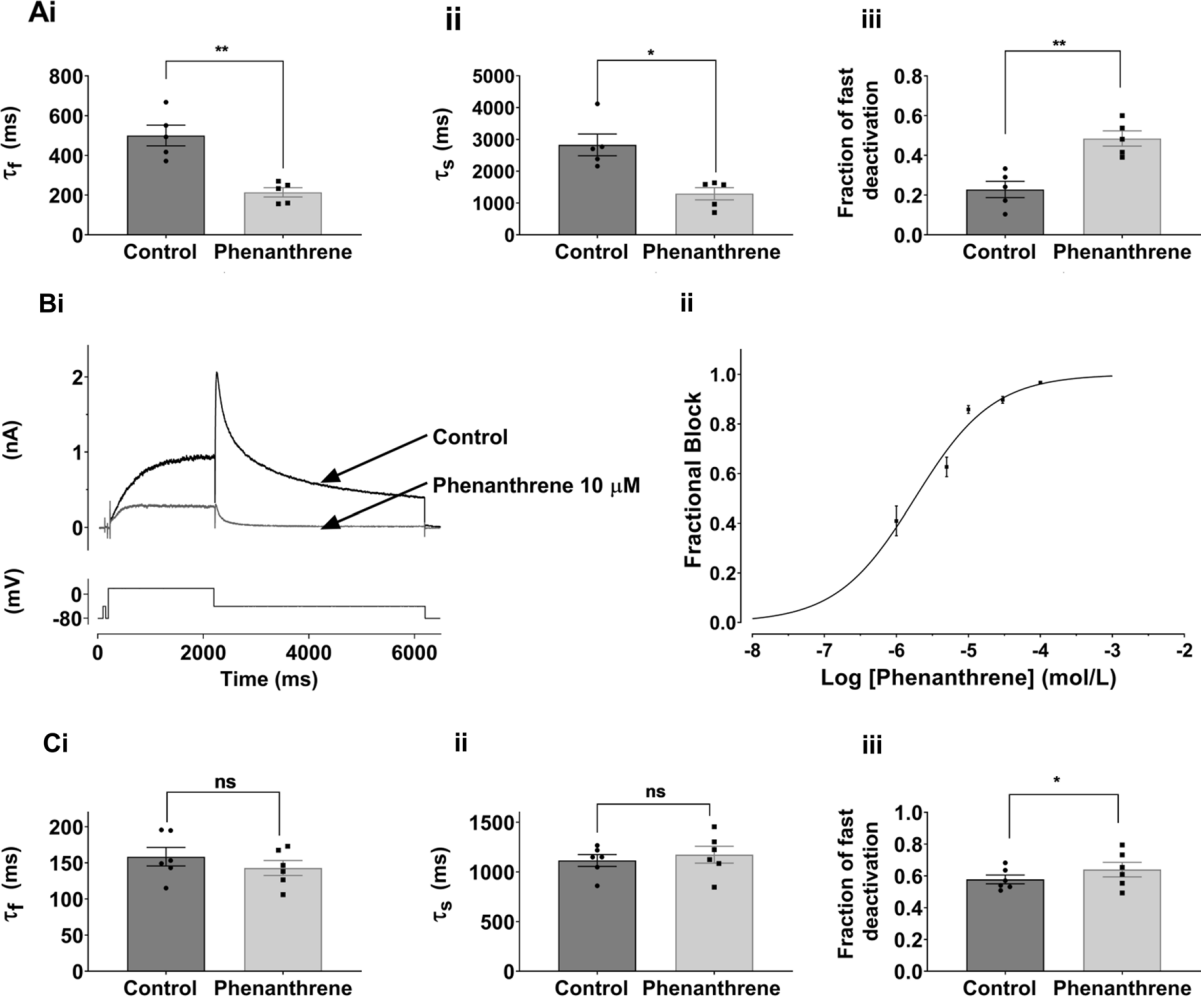
**A** Bar charts comparing deactivation parameters for I_hERG_ (hERG1a) tails elicited on repolarization to −40 mV from +20 mV in Control (black) and 10 µM phenanthrene (grey). Biexponential fitting yielded fast and slow deactivation time constants (τ_f_ and τ_s_ respectively), plotted in **Ai** and **Aii**. **Aiii** shows that the proportion of fast deactivating current increased in phenanthrene. Significance is denoted by asterisks **p < 0.01; *p < 0.05 (*n* = 5). Bar charts show mean ± SEM values, with individual experimental values in control and phenanthrene superimposed as filled circles and squares respectively. **B: Bi** shows representative traces of hERG1a/1b I_hERG_ elicited by the standard protocol shown in Fig. 1 in the absence and presence of 10 µM phenanthrene. **Bii** shows concentration response data for 5 phenanthrene concentrations (1 µM, 5 µM, 10 µM, 30 µM; 100 µM; *n* = at least 5 at each concentration). The data were fitted with Eq. 2, yielding a half-maximal inhibitory concentration (IC_50_) 1.8 ± 0.3 µM and Hill coefficient of 0.80 ± 0.09. **C** Bar charts showing deactivation parameters for I_hERG_ (hERG1a/1b) tails elicited on repolarization to -40 mV from +20 mV in Control (black) and 1 µM phenanthrene (grey). Biexponential fitting yielded fast and slow deactivation time constants (τ_f_ and τ_s_ respectively), plotted in **Ci** and **Cii**. **Ciii** shows that the proportion of fast deactivating current increased in the presence of phenanthrene (*p < 0.05 *n* = 6). Bar charts show mean ± SEM values, with individual experimental values in control and phenanthrene superimposed as filled circles and squares respectively

Experiments were performed on hERG1a current (I_hERG1a_) except for the data in Fig. 2B, C, which were conducted using co-expressed hERG1a and 1b channels (I_hERG1a/1b_) and Supplementary Fig. 2 which shows data from zERG (I_zERG_).

### Electrophysiological recording

Recordings were made as described previously [25, 26]. In brief, electrophysiological recordings were made using an Axopatch 200B amplifier (Molecular Devices) with a CV-4/100 headstage and data acquisition via a Digidata 1320 interface (Molecular Devices). Glass shards with plated HEK293 cells were placed in the recording chamber of an inverted microscope (Nikon Diaphot, USA). The extracellular superfusate was a standard Tyrode’s solution containing (in mM): 140 NaCl, 4 KCl, 2.0 CaCl_2_, 1 MgCl_2_, 10 glucose and 5 HEPES (titrated to pH 7.4 with NaOH) [24–26]. Patch pipettes (AM systems Inc, USA) had resistances of 2–4 MΩ and were filled with a solution containing (in mM): 130 KCl, 1 MgCl_2_, 5 EGTA, 5 MgATP and 10 HEPES (titrated to pH 7.2 with KOH) [24–26]. Series resistance was typically compensated by 60–80%. Currents were filtered at 1–5 kHz depending on the voltage protocol used and were digitized at 10 kHz. Except for online Supplementary Fig. 3A, all measurements were made at room temperature (21 ± 1 °C) and run-down correction was not performed. Room temperature was employed to facilitate accurate evaluation of phenanthrene effects because in pilot experiments at 37 °C, stability of phenanthrene-containing superfusate appeared variable. It also facilitated comparison of phenanthrene blocking potency between human and zebrafish ERG data in this study and with prior recordings from fish myocytes conducted at room temperature [11–13].

### Trafficking assay

Evaluation of the effects of 3 and 30 µM phenanthrene on hERG channel trafficking was performed using a *LI-COR* based In-Cell/On-Cell Western assay, as described previously [32] (see online supplement for more details).

### Phenanthrene

Phenanthrene was obtained from Merck (Sigma-Aldrich) and dissolved in DMSO to give stock solutions of 20, 30 and 50 mM. Experimental solutions with phenanthrene concentrations shown in the Results contained a maximum of 0.1% DMSO.

### Computational docking

Docking of phenanthrene to hERG was initially performed using the cryo-EM derived structure for hERG [33] (PDB code 5VA2), as described previously [26, 29, 34]. A model closely related to the cryo-EM structure was obtained from a short molecular dynamics (MD) simulation in which the F656 side chain of one of the four hERG subunits was found to reorient towards the pore—this subunit was then replicated around all four pore subunits to produce a model with all four F656 side chains facing the pore; this structure was used for the docking data shown in Fig. 6. The phenanthrene structure was converted from SMILE representation (obtained from PubChem database) to a 3D structure, hydrogens were added and the molecule energy minimised.

Phenanthrene was docked in the hERG structure and in the related model using GOLD (GOLD version 5.6; Cambridge Crystallographic Data Centre, Cambridge, UK). The central pore cavity was initially chosen as a binding site where a radius of 10 angstrom extended from the centre of the cavity and in a level with a middle point between the canonical aromatic residues F656 and Y652. The side chains of these aromatic residues were set to be freely flexible during docking simulations. Rotamer sampling was maximally set to 300,000 generations. Dockings were scored by Goldscore and rescored by Chemscore. Two hundred docking repeats were made in each case and the low-energy-score poses were selected and inspected. Phenanthrene was also docked within a side pocket under the selectivity filter in the open pore F656-rotated hERG model. This binding pocket was centred above the β-carbon of Y652 and encompassed a volume having a radius of 7 angstrom. Within this selection, the side chains for the following residues from subunit A of the tetrameric channel were permitted to rotate freely: F557, L622, T623, S624, L650, M651, Y652, I655. F656 side chains from adjacent subunits (A and B) of the channel were also allowed to rotate freely. Similar setting and parameters were used as above where also 200 docking repeats for each drug were generated and low energy poses identified. In all docking runs the maximal side chain rotamer sampling allowed in GOLD was used (free rotation of 10 selected residue side chain as well as sampling of Ser, Thr and Tyr side chain hydroxyl rotamers to optimize hydrogen bond interactions) with the specific set of flexible residues chosen to match the specific binding location tested.

The results were presented using PyMOL Molecular Graphics System, Version 2.0 Schrödinger, LLC.

### Data presentation and analysis

The data are presented as mean ± SEM of the number of independent experiments indicated (*n*). Statistical comparisons were made using a Student’s *t* test, one- or two-way analysis of variance (ANOVA) followed by a Bonferroni or Dunnett’s post test, as appropriate. *P* values < 0.05 were considered to be statistically significant.

Fractional block of hERG current (I_hERG_) by the different phenanthrene (Phen) concentrations studied was determined using the equation:

1 $${\text{Fractional block}} = 1 - \left( {{{{{\text{I}}_{{{\text{hERG}} - {\text{Phen}}}} } } \mathord{\left/ {\vphantom {{\left( {{\text{I}}_{{{\text{hERG}} - {\text{Phen}}}} } \right)} {{\text{I}}_{{{\text{hERG}} - {\text{Control}}}} }}} \right. \kern-\nulldelimiterspace} {{\text{I}}_{{{\text{hERG}} - {\text{Control}}}} }}} \right)$$

where “Fractional block” refers to the degree of inhibition of hERG current by a given concentration of phenanthrene. I_hERG-Phen_ and I_hERG-Control_ represent current amplitudes in the presence and absence of phenanthrene.

Concentration–response data were fitted by a standard Hill equation of the form:

2 $${\text{Fractional block}} = {{1} \mathord{\left/ {\vphantom {{1} {\left( {{1} + \left( {{{{\text{IC}}_{{{5}0}} } \mathord{\left/ {\vphantom {{{\text{IC}}_{{{5}0}} } {\left[ {{\text{Phen}}} \right]}}} \right. \kern-\nulldelimiterspace} {\left[ {{\text{Phen}}} \right]}}} \right)^{{\text{h}}} } \right)}}} \right. \kern-\nulldelimiterspace} {\left( {{1} + \left( {{{{\text{IC}}_{{{5}0}} } \mathord{\left/ {\vphantom {{{\text{IC}}_{{{5}0}} } {\left[ {{\text{Phen}}} \right]}}} \right. \kern-\nulldelimiterspace} {\left[ {{\text{Phen}}} \right]}}} \right)^{{\text{h}}} } \right)}}$$

where IC_50_ is [Phen] producing half-maximal inhibition of the I_hERG_ tail and *h* is the Hill coefficient for the fit.

Half maximal activation voltages for I_hERG_ were obtained from current–voltage (I–V) relations from I_hERG_ tails measured at −40 mV in the absence or presence of phenanthrene following voltage commands to different test potentials, using the following Boltzmann equation:

3 $$\text{I}=\text{I}_{{\rm max}}/\left(1+\text{exp}((\text{V}_{0.5}-\text{V}_{{\rm m}})/k)\right)$$

where I = I_hERG_ tail amplitude following test potential V_m_, I_max_ is the maximal I_hERG_ tail observed during the protocol, V_0.5_ is the half maximal activation voltage of I_hERG_ and *k* is the slope factor describing I_hERG_ activation.

Voltage-dependent activation curves were constructed by calculating activation variables at 2 mV intervals between −80 mV and +40 mV. Values for V_0.5_ and *k* were derived from experimental fits to I–V data using Eq. (3) were inserted into the following equation:

4 $${\text{Activation parameter }} = \, {{1} \mathord{\left/ ({\vphantom {{1} {{1} + {\text{exp}}\left( {{{\left( {{\text{V}}_{{0.{5}}} - {\text{V}}_{{\text{m}}} } \right)} \mathord{\left/ {\vphantom {{\left( {{\text{V}}_{{0.{5}}} - {\text{V}}_{{\text{m}}} } \right)} k}} \right. \kern-\nulldelimiterspace} k}} \right)}}} \right. \kern-\nulldelimiterspace} {{1} + {\text{exp}}\left( {{{\left( {{\text{V}}_{{0.{5}}} - {\text{V}}_{{\text{m}}} } \right)} \mathord{\left/ {\vphantom {{\left( {{\text{V}}_{{0.{5}}} - {\text{V}}_{{\text{m}}} } \right)} k}} \right. \kern-\nulldelimiterspace} k}} \right)}})$$

where the ‘activation parameter’ at test potential V_m_ lies between 0 and 1 and V_0.5_ and *k* have the meanings described above for Eq. 3.

## Results

### *WT I*_*hERG*_* inhibition by phenanthrene*

The sensitivity of I_hERG_ to inhibition by phenanthrene was determined using the protocol shown in Fig. 1B. This was comprised of a 2 s depolarization from −80 mV to +20 mV, followed by repolarization to −40 mV, at which the resurgent tail current that is typical of hERG was observed. I_hERG_ tail magnitude was measured relative to current elicited by a brief (50 ms) pulse from −80 to −40 mV that preceded the 2 s voltage command. This protocol has been used in multiple prior studies of I_hERG_ pharmacology from our laboratory (e.g. [24–26, 35]). Figure 1B shows exemplar records of I_hERG_ in control solution, the presence of 10 μM phenanthrene and following washout, whilst Fig. 1C shows a continuous plot of I_hERG_ tail amplitude during the experiment from which these example traces were taken. Phenanthrene application led to a progressive decline in I_hERG_ amplitude, which was largely reversible on washout. Six concentrations of phenanthrene, ranging from 1 to 100 µM, were tested in similar experiments. Higher concentrations could not be investigated due to issues with phenanthrene solubility and exposure of cells to higher levels of solvent. Fractional inhibition of I_hERG_ tails at each phenanthrene concentration was ascertained using Eq. 1 and values from different experiments at each concentration pooled to produce the concentration response plot shown in Fig. 1D. These data were fitted with Eq. 2, which yielded a half-maximal inhibitory concentration (IC_50_) of 17.6 ± 1.7 µM and Hill slope of 0.94 ± 0.09. At the highest concentration tested (100 µM), the mean level of tail current inhibition attained was 79.1 ± 2.9% (*n* = 5). It is apparent from the traces shown in Fig. 1B that in addition to reducing I_hERG_ amplitude, phenanthrene altered the deactivation time course of the I_hERG_ tail. This effect was quantified by standard biexponential fitting of deactivating I_hERG_ tails at −40 mV following 2 s duration commands to +20 mV. As shown in Fig. 2A, both fast and slow time constants of deactivation (τ_f_ and τ_s_) were significantly decreased in the presence of phenanthrene, whilst the proportion of deactivation described by τ_f_ increased. These findings indicate that phenanthrene application resulted in significant acceleration of I_hERG_ deactivation. Evaluation of the effects of chronic (24 h) treatment of 3 and 30 µM phenanthrene on hERG1a channel trafficking showed no significant reduction in surface hERG protein expression by either concentration (see online Supplementary Fig. 1). In contrast, chronic treatment with 20 µM tamoxifen significantly reduced the level of surface hERG channel expression as has been previously reported [36] (online Supplementary Fig. 1). In additional comparative experiments on zebrafish hERG (Supplementary Fig. 2), phenanthrene inhibited I_zERG_ with an IC_50_ of 8.77 ± 0.93 μM (Hill co-efficient of 0.84 ± 0.09) and accelerated I_zERG_ deactivation.

There is evidence that native mammalian I_Kr_ channels are comprised of hERG1a together with the abbreviated hERG1b isoform, which possesses a shorter and distinct N terminus and which exhibits faster deactivation compared to hERG1a [37–39]. Figure 2Bi shows the effect of 10 µM phenanthrene on co-expressed hERG1a/1b current (I_hERG1a/1b_). This concentration produced a marked reduction in I_hERG1a/1b_ amplitude. In all, five phenanthrene concentrations (1–100 µM) were tested against I_hERG1a/1b_ (Fig. 2Bii), yielding an IC_50_ value of 1.8 ± 0.3 µM (Hill coefficient 0.80 ± 0.09). At the highest phenanthrene concentration tested (100 µM) I_hERG1a/1b_ tails were inhibited by 96.7 ± 0.6% (*n* = 6). Figure 2C shows the effect of 1 µM phenanthrene on deactivation time constants of I_hERG1a/1b_. Whilst, as expected, the rate of deactivation in control solution was faster for I_hERG1a/1b_ than I_hERG1a_ alone (compare Fig. 2C with Fig. 2A), in contrast to the observed effect on I_hERG_ carried by hERG1a alone, phenanthrene application did not lead to a significant reduction in either fast or slow deactivation time constant for I_hERG1a/1b_, although the contribution of the fast component of deactivation was slightly greater in the presence of phenanthrene.

Some I_hERG_ inhibitors exhibit temperature dependence of inhibitory potency (e.g. [40, 41]). Thus, additional experiments were conducted in which the extent of hERG1a I_hERG_ tail inhibition by 10 µM phenanthrene was established at 37 °C and compared to that observed at room temperature. The results for 8 such recordings are shown in Supplemental Fig. 3A; although there was a trend towards increased I_hERG_ block at the higher temperature this did not attain statistical significance (*p* > 0.05).

### *Inhibition by phenanthrene of I*_*hERG*_* elicited at different voltages*

**Fig. 3:**
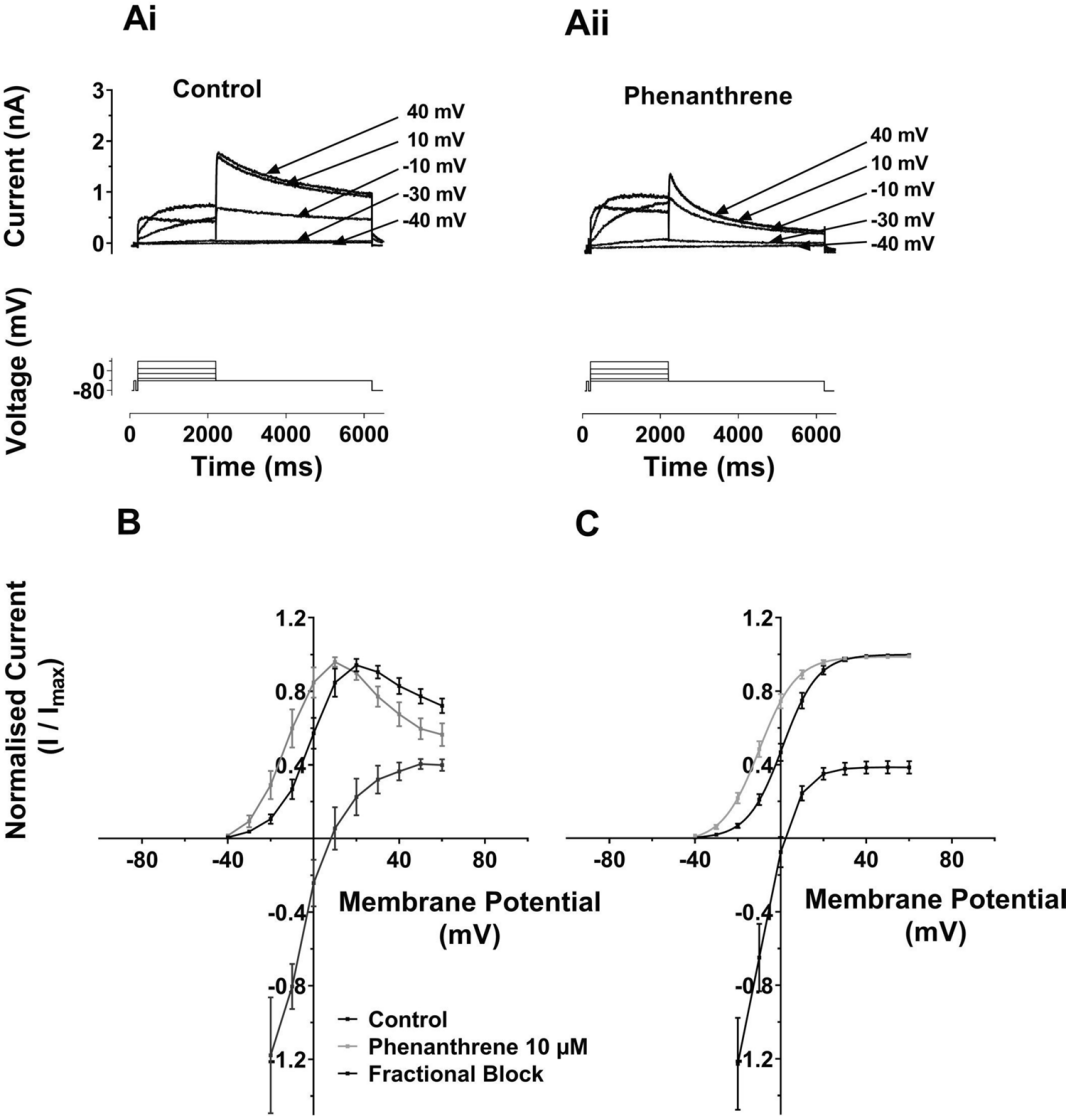
**A** Representative I_hERG_ traces (upper) are shown for **Ai**: in control solution and **Aii**: in 10 µM phenanthrene. I_hERG_ was elicited by a voltage protocol (lower traces) that was comprised of 2 s command pulses applied at 10 mV increments between −40 and +60 mV. Only selected traces are shown for clarity of display. Each current trace is labelled with its corresponding test potential. **B** Mean I–V relation for end-pulse currents. The currents were normalized to the maximum current in each of control and 10 µM phenanthrene. Corresponding mean fractional inhibition by 10 µM Phenanthrene is also shown. **C** Mean I_hERG_ tails in control (black) and phenanthrene (grey) following the different voltage commands. Currents were normalized to the maximum current in each of control and 10 µM phenanthrene Boltzmann fitting gave an activation V_0.5_ for Control of 1.3 ± 1.6 mV, slope: 7.2 ± 0.2 (*n* = 16) and for phenanthrene gave a V_0.5_ of −8.9 ± 1.5 (*p* < 0.001 vs Control), Slope: 7.8 ± 0.3 (*n* = 16)

Voltage dependence of hERG inhibition was investigated for hERG1a using the I–V protocol shown in Fig. 3Ai, ii. This protocol was similar to that used in Fig. 1, but incorporated progressively larger depolarizations (in 10 mV increments) to test potentials between −30 and +60 mV. Whilst both pulse and tail I_hERG_ with voltage commands to more positive membrane potentials in the range tested were reduced by phenanthrene (10 µM), at more negative voltages in the tested range, the current appeared to increase in amplitude in the presence of phenanthrene. Figure 3B, C shows normalized I–V relations for end-pulse current (Fig. 3B) and tail current (Fig. 3C). As Fig. 3B shows the end pulse I–V relation showed the bell-shaped characteristic of I_hERG_ in both control and phenanthrene containing solutions. Superimposed on the same voltage axes is a plot of fractional block of I_hERG_ at each voltage. As is clearly illustrated, at potentials below +10 mV facilitation (negative plotted values, representing an increase in current) of I_hERG_ was observed. This gave way to a progressive increase in end pulse current block, up to ~  +40 mV. Normalized I_hERG_ tails were plotted and fitted with Eq. 4 in Fig. 3C, along with a superimposed plot of fractional inhibition (cf [25, 35, 42]). For all test potentials negative to ~ 0 mV, facilitation (a fractional increase) in I_hERG_ tail amplitude at -40 mV was seen, whilst fractional inhibition increased markedly up to ~  +20 mV, then levelled off at positive potentials. The normalized I_hERG_ tail I–V relation exhibited a left-ward voltage shift in the presence of phenanthrene. In control solution the V_0.5_ value obtained was 1.3 ± 1.6 mV (*k* = 7.2 ± 0.2 mV, *n* = 16), whilst in 10 µM phenanthrene V_0.5_ was -8.9 ± 1.5 mV (*P* < 0.001 vs Control; *k* unchanged 7.8 ± 0.3 mV, *n* = 16). Supplementary Fig. 3B shows an alternative display format: plots of I_hERG_ tail current density. These illustrate the increase in tail current density at negative voltages and inhibition at more positive voltages in the tested range and also show the leftward shifted activation in phenanthrene. Thus, phenanthrene produced a ~ 10 mV negative shift in voltage-dependent activation associated with the facilitation of I_hERG_ at negative voltages. This phenomenon has been observed previously for other hERG-blocking agents, including azimilide [43], 4-aminopyridine [42] and sarizotan [35]. Notably, the steep change in fractional inhibition coincided with the steep portion of the voltage-dependent activation relation for I_hERG_ (Fig. 3C) consistent with an activation-dependent inhibitory mechanism.

### *Depolarization time and I*_*hERG*_* inhibition by phenanthrene*

**Fig. 4:**
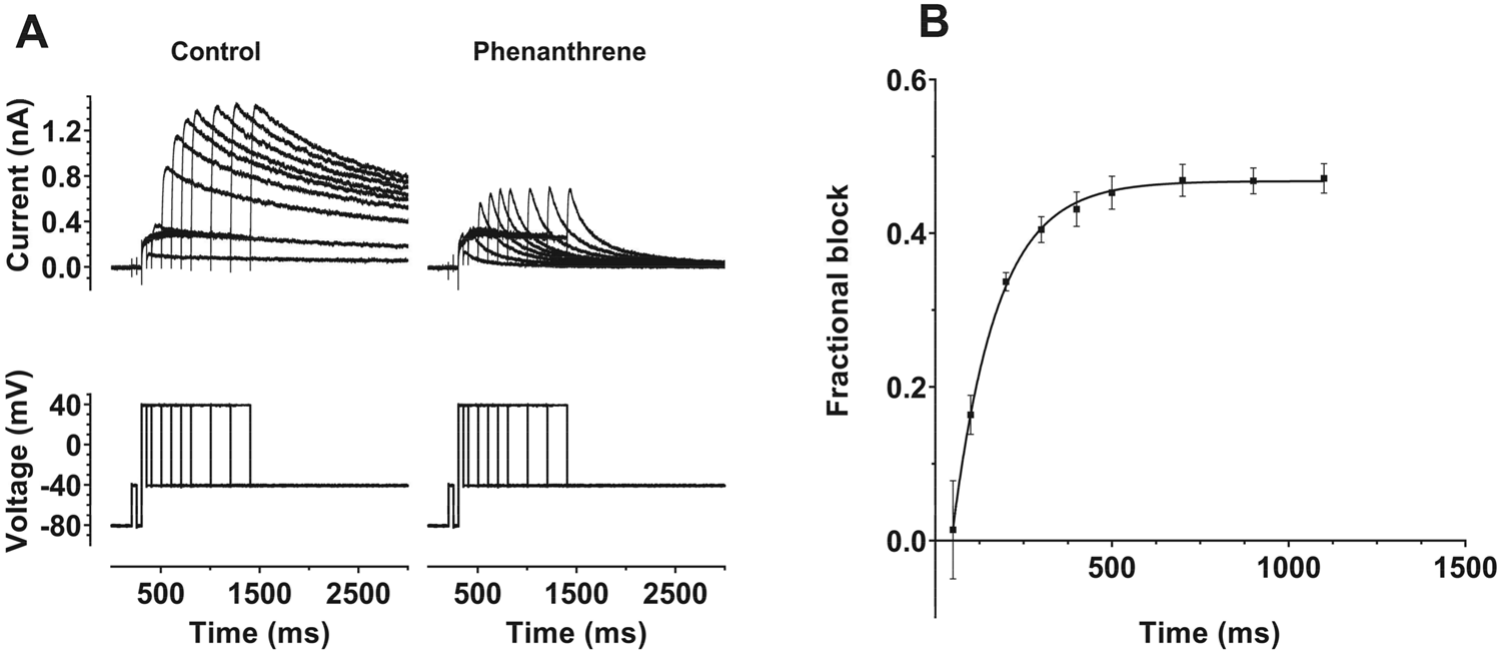
**A** Upper traces show representative I_hERG_ records elicited by protocol shown as lower traces. Tail currents were elicited at −40 mV following depolarizing pulses to +40 mV for differing time periods between 50 and 1100 ms. Left hand panel shows recordings in control solution and right-hand panel contains recordings from the same cell during exposure to 30 µM phenanthrene. **B** Plot of mean fractional block of I_hERG_ tail by 30 µM phenanthrene against command pulse duration (*n* = 5). A monoexponential fit to these data (from each experiment) gave an inhibition time constant (τ_inhib_) of 130.2 ± 15.4 ms. A one-way ANOVA showed an overall significance in the time dependence of inhibition (*p* < 0.0001), with (Bonferroni) post hoc testing showing that inhibition following pulses of up to 200 ms in duration was significantly different from all durations of 500 ms and longer (*p* < 0.05 or less for all comparisons)

The voltage dependence of I_hERG_ inhibition by phenanthrene indicates gating dependence of channel inhibition by the compound. To probe this further, we conducted experiments in which the extent of I_hERG_ inhibition by voltage commands of different durations was established [42, 44, 45]. For these experiments, activating pulses to +40 mV (a potential observed to be at the top of the voltage-dependent activation relations for control and phenanthrene) of different durations between 50 and 1100 ms were applied. These were first applied in control superfusate, the cell under study was then rested for 3–5 min whilst being superfused with 30 µM phenanthrene and the protocol was reapplied. The extent of I_hERG_ inhibition by the compound for the different duration commands was then evaluated by assessing the extent of tail current block. The left panel of Fig. 4A shows a family of current traces in control solution (current—upper traces, voltage protocol—lower traces), whilst the right panel of Fig. 4A shows corresponding data in 30 µM phenanthrene. The increase in tail current amplitude as pulse duration increased (for both conditions) reflects the progressively increased time periods for activation of I_hERG_. As these example traces indicate, there was little inhibition of I_hERG_ following brief depolarizations, but inhibition increased with command pulse duration. The mean fractional block data are plotted against command pulse duration in Fig. 4B, showing a clear time dependence of inhibition, with block increasing markedly as the command pulse increased between 50 and 500 ms, levelling out at longer pulse durations in the range tested. Thus, there was clear dependence of the response to phenanthrene on the duration of the activating test command (*p* < 0.0001 one-way ANOVA; *n* = 5). A monoexponential fit to the data in Fig. 4B yielded a time constant for development of inhibition τ_inhib_ of 130.2 ± 15.4 ms. The increase in tail current inhibition as command pulse duration lengthened was associated with an apparent increase in rate of development of I_hERG_ activation at +40 mV, with τ_act_ values of 123.3 ± 75.6 ms and 75.6 ± 9.8 ms, respectively, in control and phenanthrene (*p* < 0.01). The results of this experiment are consistent with a clear dependence of phenanthrene inhibition of I_hERG_ on channel gating, with little or no inhibition of closed channels evident from the plot in Fig. 4B.

### *Probing the mechanism of I*_*hERG*_* inhibition with mutagenesis*

**Fig. 5:**
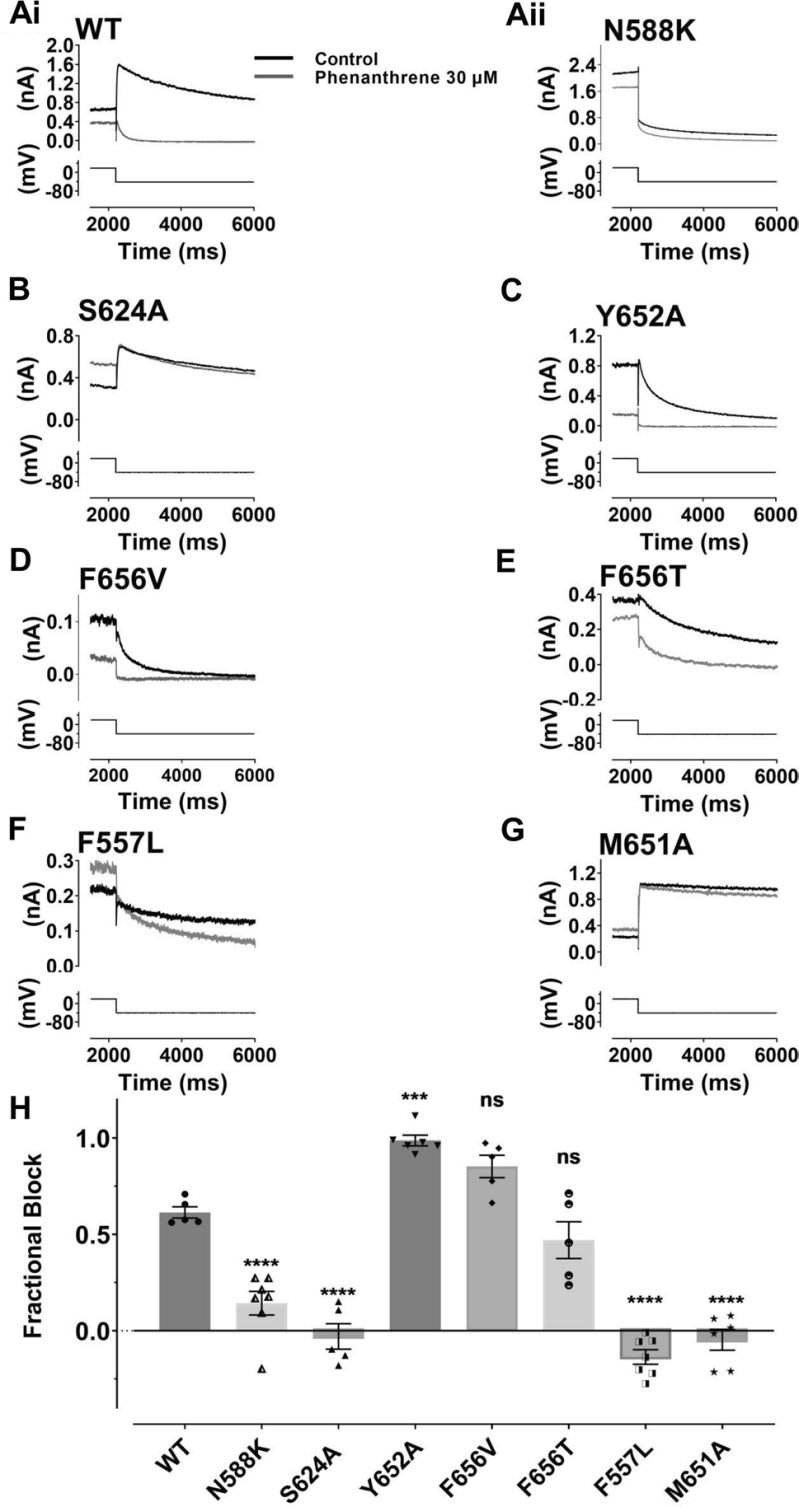
Effect of hERG mutations on the inhibitory effect of phenanthrene on I_hERG_. **A–G** show representative tail current traces (upper) for control I_hERG_ (black) and during application of 30 µM Phen I_hERG_ (grey), elicited by same voltage protocol used for Fig. 1 (lower) as described. **Ai**: wild type (WT), **Aii**: N588K, **B** S624A, **C** Y652A, **D** F656V, **E** F656T, **F** F557L and **G** M651A. **H** Bar chart shows extent of mean tail current block by 30 µM for WT, N588K, Y652A, S624A, F656V, F656T, F557L and M651A I_hERG_ respectively. One way ANOVA (*F* = 61.2, *p* < 0.0001). Significance for post hoc Bonferroni comparisons of each mutant with WT is denoted by asterisks ****p < 0.0001, ***p < 0.001 (WT *n* = 5, N588K *n* = 7, Y652A *n* = 6, S624A *n* = 5, F656V *n* = 5, F656T *n* = 5, F557L *n* = 7 and M651A *n* = 6). Bar charts show mean ± SEM values, with individual experimental values for each mutant superimposed as the different symbols overlying the plotted bars

In order to investigate gating dependence of inhibition further, experiments were conducted using the N588K attenuated-inactivation mutation. The N588 residue is located in the external S5-Pore linker region of the channel; it has utility for investigating inactivation dependence of hERG inhibition [46, 47] as it perturbs inactivation gating without directly modifying channel structure within regions of the pore typically implicated in drug binding. Figure 5Ai shows the effect of 30 µM phenanthrene on WT I_hERG_, illustrating marked current inhibition by this concentration of the compound. In contrast, only slight inhibition of N588K I_hERG_ was observed (Fig. 5Aii, mean data in Fig. 5H). This indicates that inactivation competency was required for optimal interaction between phenanthrene and its binding site on the hERG channel. The S624 residue, located near the base of the hERG channel selectivity filter, has been implicated in I_hERG_ inhibition by a number of compounds (e.g. [48, 49]). Figure 5B shows effects of 30 µM phenanthrene on S624A I_hERG_. Pulse current appeared significantly augmented and tail current amplitude unchanged by phenanthrene with the S624A mutation (mean data are shown in Fig. 5H). This implicates the S624 residue, directly or indirectly, as being important for phenanthrene binding to the hERG channel. We proceeded to investigate the roles of two aromatic residues, Y652 and F656 in phenanthrene action. For virtually all compounds that have been investigated in detail, interactions with one or both of these residues are obligatory for drug binding and channel inhibition [17, 50]. Remarkably, the inhibitory effect of 30 µM phenanthrene on Y652A I_hERG_ (Fig. 5C, mean data in Fig. 5H) was significantly greater than on WT I_hERG_. Supplemental Fig. 4A shows mean concentration–response data for Y652A I_hERG_, which yielded an IC_50_ of 0.46 ± 0.01 µM, Hill slope: 0.58 ± 0.11 (at the highest phenanthrene concentration tested (30 µM) I_hERG_ tails were inhibited by 98.7 ± 2.6%; *n* = 7). The F656V mutation (Fig. 5D, mean data in Fig. 5H) also did not impair the inhibitory effect of phenanthrene, showing a trend towards increased block, albeit that this was statistically insignificant. We also investigated effects of a second mutation at F656:F656T. A small reduction in inhibition of F656T I_hERG_ by 30 µM is shown in the exemplar traces shown in Fig. 5E; however, mean pooled data (Fig. 5H) show that this effect was not statistically significantly different from block of WT I_hERG_. Phenanthrene is therefore distinct from most hERG-blocking drugs thus far studied in not requiring aromatic residues at positions 652 and 656 for inhibition to occur. Residue Y652 has previously been implicated in voltage dependence of I_hERG_ inhibition (e.g. [51, 52]). In this respect, it is notable that whilst the Y652A mutation increased rather than decreased phenanthrene inhibition, it largely abolished the voltage dependence of inhibition seen for WT I_hERG_ (Supplemental Fig. 4B).

Residue F557 on the S5 helix of the hERG channel has been identified as a determinant of I_hERG_ inhibition and lies adjacent to Y652 on the S6 helix [29, 50, 53, 54]. No inhibition of F557L I_hERG_ was observed with 3 concentrations of phenanthrene (between 10 and 100 µM). Figure 5F shows representative traces of effects of 30 µM phenanthrene on F557L I_hERG_. No inhibition was evident, rather the pulse and peak tail current were slightly larger in the presence of phenanthrene than in control solution (mean data shown in Fig. 5H). This observation implicates residue F557 as of critical importance to phenanthrene inhibition of I_hERG_. The publication of a cryo-EM structure for hERG has shown the presence of 4 hydrophobic pockets that surround the central cavity of the channel, which may provide interaction site(s) for drugs [33]. Lipophilic drug access to the channel pore involving F557 and S6 residue M651 has recently been suggested for the bradycardic agent ivabradine [54]. Figure 5G shows effects of the M651A mutation on I_hERG_ block by 30 µM phenanthrene. In contrast to WT I_hERG_ M651A was little affected by phenanthrene (mean data shown in Fig. 5H), implicating this residue in the compound’s inhibitory action.

### Phenanthrene inhibition explored through computational docking

Low energy score docking poses consistent with mutagenesis data were not obtained for phenanthrene bound into the structure of hERG obtained from cryo electron microscopy (cryo-EM). Phenanthrene was unable to make multiple interactions with aromatic side chains when docked into the canonical pore blocker site below the selectivity filter within the K^+^ permeation path (not shown). This results from the absence of F656 side chain conformations in the cryo-EM structure that project the side chains towards the pore and the rigidity of the phenanthrene tri-aromatic ring structure that limits its ability to conform to the arrangement of Y652 side chains within the pore. The rigidity of the structure also limited identification of low energy score configurations for phenanthrene binding within one of the hydrophobic pockets below the pore helix in the cryo-EM structure. In addition, the projection of the F656 side chains towards the S5 helix in the cryo-EM structure limits the localisation of phenanthrene in a configuration that allows interactions with the side chains of F557 and M651.

**Fig. 6:**
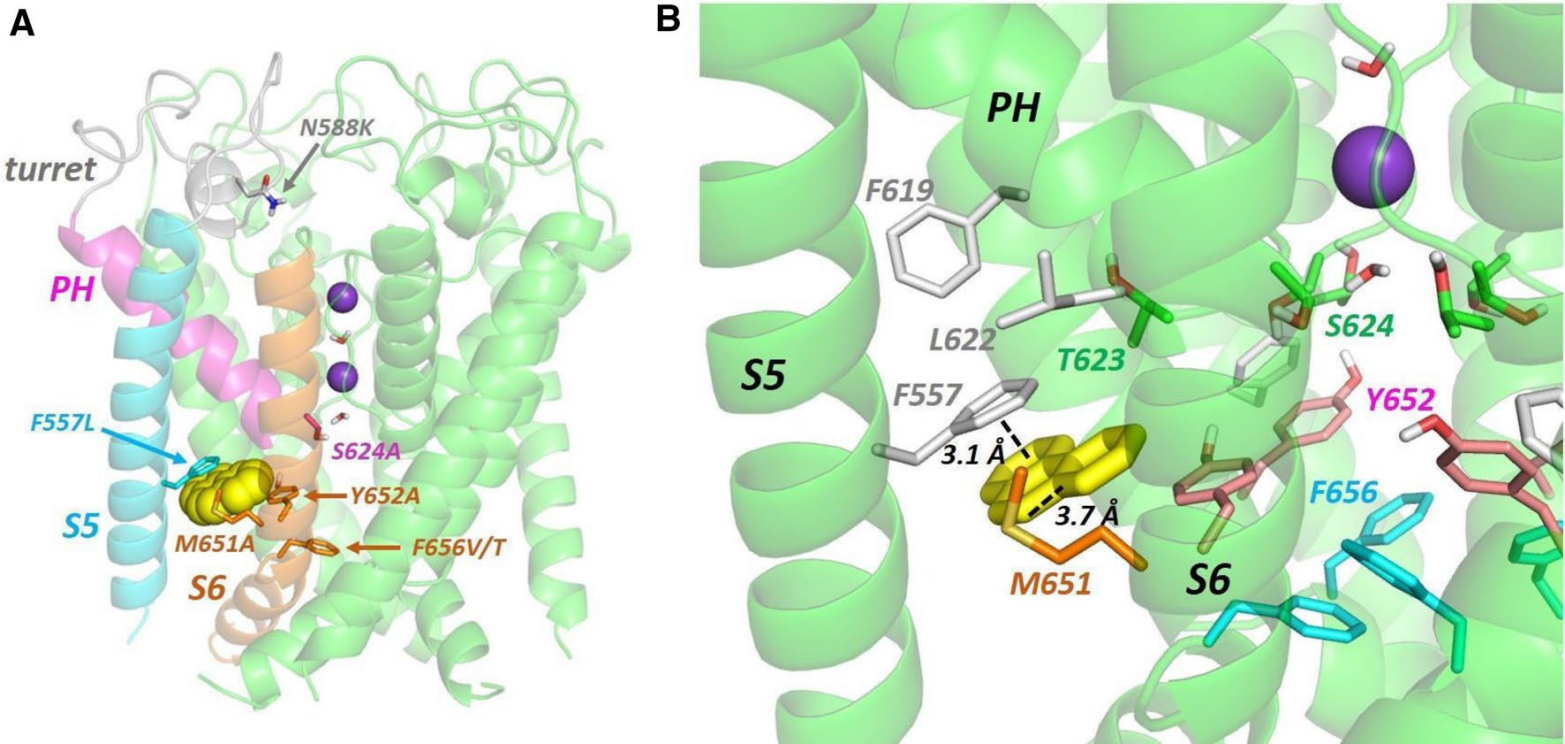
**A** Low energy score pose for phenanthrene (yellow space filling representation) docked into the hERG pore. The hERG subunit with docked phenanthrene shows the location within a single subunit of the mutations described in the text; the structural elements are coloured to match the alignment in Fig. 1a. Purple spheres are K^+^ ions in the 1 and 3 positions of the selectivity filter. **B** Close-up of a hydrophobic pocket containing docked phenanthrene (yellow sticks) in the same pose as panel **A**. An aromatic pi-stacking interaction with the F557 side chain and close interactions with the M651 side chain are indicated with dotted lines

Low energy poses were obtained for phenanthrene bound within a side pocket of a hERG model obtained by very short MD simulation in which an F656 side chain rotated away from its configuration in the cryo-EM structure towards the pore. A low energy score pose consistent with the mutagenesis data is shown in Fig. 6. Phenanthrene sits deep within a hydrophobic pocket making aromatic stacking interactions with F557 and hydrophobic interactions with M651. This binding mode is facilitated by the rotation of the F656 side chain out of this binding pocket.

## Discussion

### *Potency of observed I*_*hERG*_* inhibition and environmental relevance for human health*

Phenanthrene has previously been reported to inhibit native I_Kr_ from bluefin tuna myocytes at room temperature by nearly 60% at 5 µM and by over 80% at 25 µM [11]. 30 µM phenanthrene inhibited rainbow trout myocyte I_Kr_ by 79% [13], whilst an IC_50_ for inhibition of brown trout myocyte I_Kr_ of 7.2 µM has been reported [14]. Very recent work has indicated that I_Kr_ from zebrafish ventricular myocytes is inhibited by phenanthrene with an IC_50_ of 3.3 µM [15], which is in fair agreement with the value of 8.7 µM obtained in this study for I_zERG_. We observed IC_50_ values for I_hERG_ carried by hERG1a channels of 17.6 µM and for co-expressed hERG1a/1b of 1.8 µM, which are broadly comparable to the potency of phenanthrene against fish I_Kr_. It is notable that for I_hERG_ 1a/1b, which is thought to underlie native mammalian I_Kr_ [37–39], the maximal level of observed I_hERG_ tail inhibition (at 100 µM) was near complete (~ 97%). For I_hERG_ carried by hERG1a alone, the maximal level of observed tail current inhibition (at 100 µM) did not exceed 80%. Although the result for I_hERG1a/1b_ (and also that for Y652A on a hERG 1a background) indicates that complete or near complete channel inhibition by phenanthrene is possible, we cannot entirely exclude the possibility that maximal possible attainable inhibition of WT hERG1a I_hERG_ may be < 100%. However, due to solubility concerns it was not possible to test phenanthrene concentrations higher than 100 µM.

Data on plasma levels of PAHs in humans are sparse, in part because convention focuses on other measures—either PAH concentrations per given volume of air or urinary metabolite concentrations. Plasma concentrations of phenanthrene and/or other PAHs in the nM range have been reported in the literature (e.g. [55, 56]). However, as has been highlighted elsewhere, phenanthrene is highly lipophilic and so is likely to exhibit tissue accumulation [1]. This has been demonstrated for waterborne exposures of developing fish embryos in which phenanthrene tissue levels typically reach the low micromolar range (e.g. [57]) and in similarly substantial accumulations of PAHs in avian tissues [58]. Road pavers and coke plant workers show urinary phenanthrol levels of ~ 35–686 µg/L (approximately 0.2–3.5 µM; [59]). Such data are suggestive of quite high concentrations in subpopulations who, through occupational risk, are exposed routinely to high levels of PAHs. Moreover, a recent comparison of serum phenanthrene levels in Greek citizens found increased levels in those with heart failure (231 µg/L; ~ 1.3 µM) compared to those without heart failure (56.5 µg/L; 314 nM) [60]. Thus, it is likely to be those with high PAH exposure and/or those with pre-existing cardiovascular morbidity who will be most susceptible to adverse reactions to phenanthrene.

### Evidence for a direct channel interaction

Phenanthrene has a simple structure of three fused benzene rings and given its high lipophilicity [1], a natural question is whether it exerts direct interactions with the hERG channel or whether its inhibitory effect is secondary to accumulation of phenanthrene in membrane lipid? Multiple lines of evidence provided here support a direct interaction with the channel. First, the lack of significant effect of phenanthrene on hERG trafficking at 3 and 30 µM (Supplementary Fig. 1), indicates that altered trafficking is unlikely to contribute an additional inhibitory effect on the channel’s function during chronic exposure. Second, the structurally related antimalarial drug halofantrine (3-(dibutylamino)-1-[1,3-dichloro-6-(trifluoromethyl)phenanthren-9-yl]propan-1-ol), which contains a substituted phenanthrene, is known to inhibit I_hERG_ in an open state-dependent fashion, with sensitivity to mutation of S6 helical aromatic residues [61, 62]. Third, in this study phenanthrene inhibition of WT I_hERG_ exhibited voltage- and time dependence that demonstrate contingency on channel gating for the compound to access its inhibitory binding site. Fourth, the profound reduction in the I_hERG_ inhibitory effect of phenanthrene on inactivation-impaired N588K channels suggests a requirement for intact inactivation gating for optimal access to the inhibitory binding site. Although I_hERG_ carried by hERG1a/1b (which showed enhanced sensitivity to phenanthrene inhibition) has been reported to exhibit a modest (+28 mV) positive shift in voltage dependence of inactivation compared to WT hERG alone [39], the inactivation shift produced by the N588K mutation is much greater (+60 to +90 mV; [28, 63]) and therefore more impactful on I_hERG_ elicited by a command protocol to +20 mV, as used here to evaluate I_hERG_ inhibition. Our data suggest that the profound disturbance of inactivation gating with this mutant adversely affects channel conformation(s) favouring phenanthrene access/binding. Finally, the results of our mutagenesis experiments and docking simulations implicate specific mutations as phenanthrene binding determinants. Thus, the results of this study support a conclusion that phenanthrene acts as a direct hERG channel inhibitor. To our knowledge, the data from this study constitute the most detailed information on the basis of phenanthrene inhibition of any ion channel or electrogenic transporter.

### *Facilitation accompanying phenanthrene block of I*_*hERG*_

Phenanthrene exhibited a positive voltage dependence of WT I_hERG_ inhibition (the extent of observed inhibition increased steeply over the range of voltage-dependent activation of the channel), in common with a number of gated-state dependent small molecule inhibitors of hERG that we have studied previously (e.g. [24, 35, 42, 64, 65]). An inverse voltage dependence of I_hERG_ reduction is rarely observed, for example with protons [66, 67] or with the toxin inhibitor BeKm-1 that produces preferential closed channel inhibition [68]. Interestingly, at some voltages in the tested range, an apparent augmentation or ‘facilitation’ of I_hERG_ by phenanthrene was observed. The issue of I_hERG_ facilitation by several inhibitors has been investigated previously by Kurachi and colleagues [69–71]. These authors attributed I_hERG_ facilitation at low voltages by amiodarone, carvedilol, nifekalant and quinidine to hyperpolarization of the voltage dependence of I_hERG_ activation [69]. Remarkably, the left-ward shift in I_hERG_ activation V_0.5_ by amiodarone, carvedilol, nifekalant and quinidine did not show concentration dependence for any of these compounds [69]. Our experiments have shown that phenanthrene also negatively shifts WT I_hERG_ activation (Fig. 3). Although we did not evaluate concentration dependence of the activation V_0.5_ shift for the WT channel, it is notable that we found that Y652A I_hERG_ did not exhibit current facilitation by phenanthrene at negative voltages. This corresponded with a lack of negatively shifted activation with phenanthrene for the Y652A mutant (supplemental Fig. 4). Thus, the observed facilitation of WT I_hERG_ by phenanthrene at negative test potentials can reasonably be attributed to the leftward shift in voltage dependent activation. A pharmacophore for hERG channel facilitation by inhibitors has been proposed that is comprised of one positively ionizable feature and 3 hydrophobic features [71]. Two of the facilitator molecules used in construction of this pharmacophore were nortryptiline and imipramine, both of which are comprised of 3 rings fused together with a side chain [71]. Phenanthrene is chemically simpler than either of these 3 ringed molecules, possessing 3 fused rings, but lacking an ionizable feature/size chain. Our results therefore indicate, at least in the case of phenanthrene, that 3 hydrophobic features alone can suffice for I_hERG_ facilitation to occur. It has been recently suggested that facilitation of native I_Kr_ by some hERG channel inhibitors may suppress proarrhythmic early afterdepolarizations [70]. Whether or not this might be the case for phenanthrene would require direct study using ventricular myocytes from an appropriate mammalian species. In zebrafish myocytes, however, the combination of the compound’s ability to inhibit I_Kr_ and to accelerate deactivation led to a reduction in the native channel’s ability to generate protective outward I_Kr_ transients in response to premature stimulation late in repolarization/early in diastole [15].

### *Phenanthrene inhibition of I*_*hERG*_*–notable and unusual features.*

The acceleration of WT I_hERG(1a)_ and I_zERG_ deactivation seen here by phenanthrene is unusual, although it is in agreement with recent observations on the effect of phenanthrene on zebrafish native I_Kr_ [15]. Acceleration of I_hERG_ deactivation precludes a “foot-in-door” inhibitory mechanism in which a compound’s presence in the inner cavity slows closure of the activation gate. The relationship between deactivation acceleration by phenanthrene and the compound’s ability to block the channel appears complex: both N588K and S624A I_hERG_, which were largely resistant to phenanthrene inhibition, showed little or no effect of the compound on deactivation parameters (Supplemental Fig. 5), consistent with a link between current blocking and deactivation accelerating actions. On the other hand, F557L current was augmented not inhibited by phenanthrene and yet exhibited faster deactivation than in control (Fig. 5 and online supplement text), consistent with independence between current blocking and deactivation accelerating actions. It is not straightforward to unify these observations, though the F557L result indicates that deactivation acceleration is not inextricably linked to channel block by phenanthrene. There was, however, a strong correlation between the current’s deactivation rate in *control* solution and the derived IC_50_ for phenanthrene inhibition (shown by plotting IC_50_ against fast and slow inactivation τ for hERG1a, hERG1a/b and zERG; see Supplemental Fig. 6). hERG1b is identical to hERG1a except for its abbreviated N terminus, which lacks the EAG domain that is known to be important for slow deactivation (Supplemental Fig. 6 and [72]). The faster deactivation of I_hERG1a/1b_ in control conditions was not further accelerated by phenanthrene, suggesting that a full complement of intact N-termini was not required for phenanthrene to inhibit hERG channels. However, it seems likely that conformational changes associated with fast deactivation may be involved in optimising the compound’s interaction with or access to binding residues in the pore.

Perhaps the most surprising results of this study are those pertaining to the likely interaction site through which phenanthrene inhibits I_hERG_. Virtually all compounds that have been hitherto investigated have shown a strong dependence on interactions with one or both of S6 Y652 and F656: mutations of these residues typically reduce inhibitory potency [18, 50]. Indeed, structurally related halofantrine conforms to this observation [62]. In contrast, for phenanthrene, mutations at F656 failed significantly to reduce I_hERG_ inhibition and the Y652A mutation increased blocking potency. Although Y652A has previously been associated with a modest (+33 mV) positive shift in the voltage dependence inactivation [73], the extent of I_hERG_ inactivation at +20 mV (the command voltage used in our standard I_hERG_ protocol) was shown to be similar between WT and Y652A I_hERG_ [73]. Consequently, differences between WT and Y652A inactivation would not have been a significant factor in experiments investigating phenanthrene with this protocol. To our knowledge there is only one prior report of a compound for which Y652A increases inhibitory potency: capsaicin [74]. In that study, the IC_50_ for capsaicin was four-fold lower for Y652A than for WT I_hERG_ and the F656A mutation did not alter potency [74]. Obligatory determinants of capsaicin binding were not identified [74]. There is little structural similarity between capsaicin and phenanthrene and binding determinants are unlikely to be similar for the two compounds. The recent cryo-EM structure of hERG contains hydrophobic pockets surrounding the central cavity that are large enough to accommodate drug molecules [33]. F557 on the S5 helix lies deep within the pockets and has been implicated in the binding of a number of drugs [29, 35, 53, 75]. The recent work has implicated both F557 and M561 in hERG block by the bradycardic agent ivabradine, with mutation of M651 influencing state-dependent dynamics of aromatic residue cassettes at the pore–lipid interface [75]. These observations are consistent with the recent identification with other K^+^ channels that hydrophobic pockets can provide inhibitor binding sites away from the K^+^ permeation path [76–78].

Docking of phenanthrene identifies a site within the side pockets that can accommodate phenanthrene in structures in which the F656 side chain is rotated towards the pore. In other words, the reconfiguration of F656 into a pore-facing orientation creates space for phenanthrene to bind into a site in which interactions with F557 and M651 side chains are made (Fig. 6), consistent with our mutagenesis results (Fig. 5). Recent simulations starting with the cryo-EM structure have indicated that rotation of the F656 side chain towards the pore occurs readily and is required for optimal matching of computational analysis of drug binding with experimental mutagenesis data [29, 75, 79, 80], although in a recent bound structure for astemizole, direct interactions of the drug with F656 made only a minor contribution to overall binding [81]. Our results suggest that rotation of F656 side chains towards the pore may be required both for binding of some classical pore blockers (to maximise pore blocker interactions with aromatic side chains within the pore) and for inhibitors that bind deep within hERG pore domain side pockets (to free space for inhibitor binding). In any case, binding modes that accommodate phenanthrene deep within a pocket on the S5-S6 interface (Fig. 6) are also consistent with a minimal effect of F656 mutants on phenanthrene block (Fig. 5), in contrast with a general requirement for F656 as a binding determinant for positively charged pore blockers [50]. The location of the binding site away from the K^+^ permeation path below the selectivity filter is also consistent with the absence of effect of inward K^+^ current which can suffice to attenuate the effects of blockers that bind within the K^+^ permeation path [82, 83].

The enhancement of phenanthrene block in the Y652A mutant is less readily explained by the docking analysis although the absence of a requirement for Y652 interactions is consistent with the location for phenanthrene identified by docking guided by mutagenesis (Fig. 6). It is notable that the voltage dependence of I_hERG_ inhibition was markedly altered by the Y652A mutation (supplemental Fig. 4), with substantial inhibition reached by −30 mV and then not further increasing. The voltage dependence of phenanthrene block is thus likely to be associated with a voltage-dependent reorientation of the Y652 side chain [29, 84], implicating a change on the configuration of the Y652 side chain in promoting block. One possibility is that voltage-dependent reorientation of the Y652 side chain towards the bottom of the selectivity filter in response to membrane depolarization promotes hydrogen bonding between the tyrosine phenolic hydroxyl group and the S624 side chain hydroxyl group, as observed in previous docking to hERG homology models [34] and this facilitates phenanthrene access to the binding pocket. Such an effect might explain the attenuation of phenanthrene block in the S624A mutant.

The Hill coefficients for the concentration response data for phenanthrene block of WT I_hERG_ (Figs. 1 and 2) are consistent with a lack of cooperativity which, taken together with the small size of phenanthrene, suggests that binding to a single hERG pore subunit is sufficient for channel block to occur. Although hydrophobic pocket binding has been identified for several K^+^ channel inhibitors [76–78], the mechanism by which this leads to channel inhibition remains unclear. One possibility is that inhibition involves perturbation of the selectivity filter. A curious observation is that some hERG activators which act by attenuating inactivation also bind within hERG pockets and so there may be subtle differences in blocker and activator binding that affect structure around the selectivity filter with opposite effects. Consistent with this idea a recent study on hERG activators that are likely to bind within pockets has shown that subtle chemical modification of activators can turn them into blockers [85].

In conclusion, this study demonstrates that the ubiquitous hydrocarbon pollutant phenanthrene produces an acute pharmacological block of the hERG potassium channel at concentrations compatible with tissue accumulation. We have also identified phenanthrene as a direct channel inhibitor that is mechanistically distinct from canonical hERG blockers, suggesting a broader sensitivity of this channel to adverse chemical impact than simply an off-target sensitivity to pharmaceuticals. Importantly, whilst candidate pharmaceuticals with hERG-blocking activity can be withdrawn from development, this is not the case for phenanthrene—a pervasive pollutant in air and water. As I_Kr_ is critical for human ventricular repolarization, further work is warranted to determine consequences of this effect for human ventricular electrophysiology and arrhythmia risk and whether tricyclic pollutants contribute to the morbidity and mortality associated with urban air pollution.

## Supplementary Information

Below is the link to the electronic supplementary material.

Supplementary file1 (DOCX 2906 KB)

## Data Availability

The data for the study are included in the manuscript and supplementary information. Materials for the study will be made available on reasonable request.

## Code availability

The cryo-EM structure used for this study is available at the RCSB Protein Data Bank, PDB code: 5VA2.
