# Supplementary material for: Inhibition of the hERG potassium channel by phenanthrene: a polycyclic aromatic hydrocarbon pollutant
Source: Cell Mol Life Sci. 2021 Nov 2;78(23):7899–914. doi: 10.1007/s00018-021-03967-8 (PMC8629796; doi:10.1007/s00018-021-03967-8)
Supplement: Supplementary file 1 — Supplementary file1 (DOCX 2906 KB) [file 18_2021_3967_MOESM1_ESM.docx]

**Inhibition of the hERG potassium channel by phenanthrene: a polycyclic aromatic hydrocarbon pollutant**

Ehab Al-Moubarak^1^, Holly A. Shiels^2^, Yihong Zhang^1^, Chunyun Du^1^, Oliver Hanington ^1^, Stephen C Harmer ^1^, Christopher E. Dempsey^3^, Jules C. Hancox^1 *^

^1^School of Physiology, Pharmacology and Neuroscience, University of Bristol, UK.

^2^Division of Cardiovascular Sciences, University of Manchester, UK.

^3^School of Biochemistry, University of Bristol, UK.

***Online Supplementary Information***


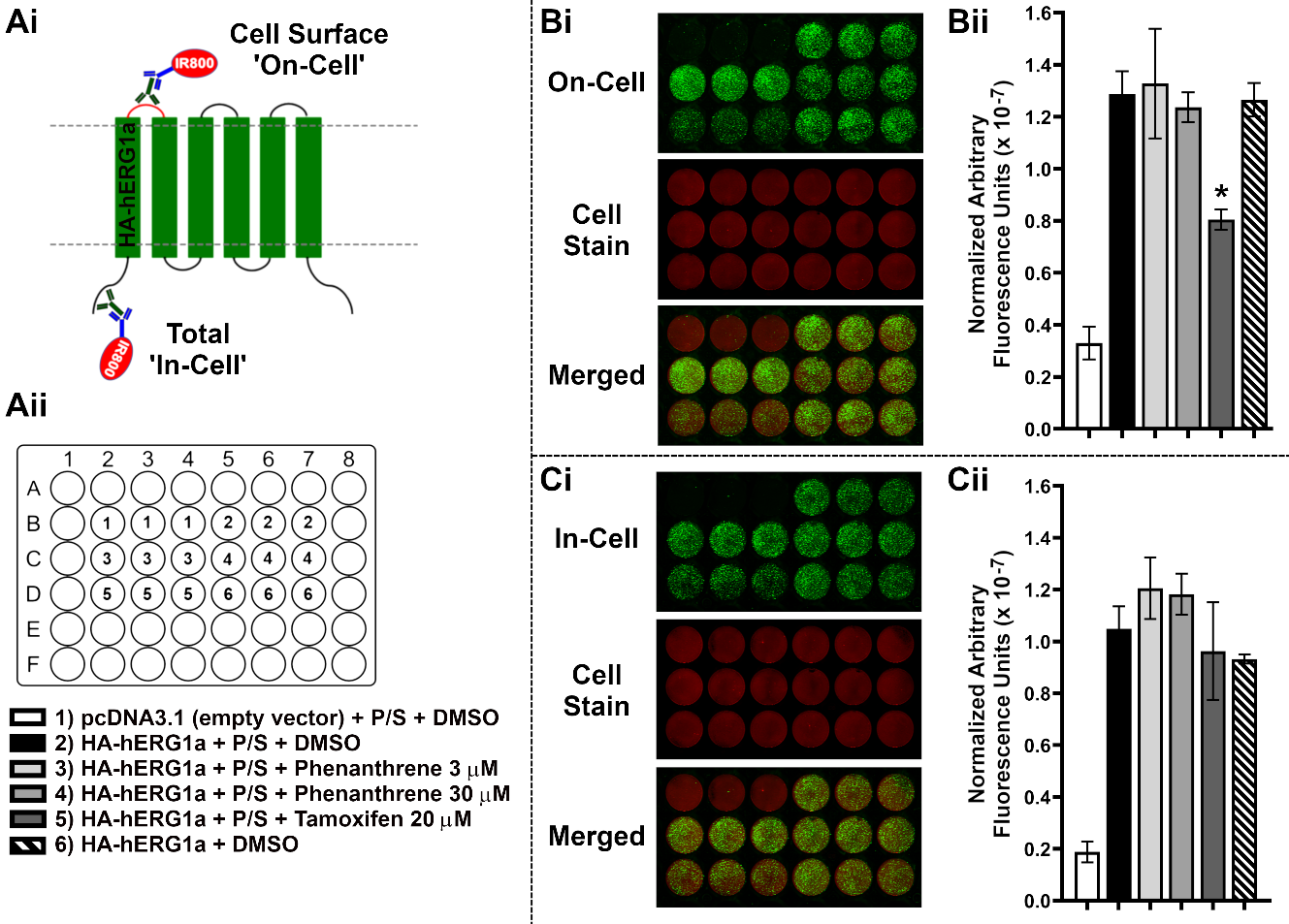


**Supplementary Figure 1:** **Effect of chronic exposure to phenanthrene on hERG1a channel trafficking.**

**Ai**) Explanatory schematic diagram for *LI-COR*^®^ based Cell Surface (‘On-Cell’) and Total cellular hERG1a expression (Total) (‘In-Cell’) Western assays. The HA epitope tag is located between S1 and S2. **Aii**) Plate layout and legend for On/In-Cell Western assays presented in panels B and C. The Methods for this assay have been described previously by Al Moubarak et al [1].

**B**) Panel **Bi** shows a representative Cell Surface (‘On-Cell’) Western assay (green channel (800); upper panel). Cell number was determined using WGA-680 (red channel (700); middle panel) and the two channels are merged in the lower panel. **Bii**: Normalized Cell Surface ‘On-Cell’ hERG1a expression levels after a 24 hour pretreatment with phenanthrene (3 or 30 μM) or tamoxifen (20 μM) when compared to control (treatment with P/S (penicillin/streptomycin) and 0.1% DMSO (dimethyl sulfoxide)).

**C**) Panel **Ci** shows a representative Total cellular (Total) (‘In-Cell’) Western assay (green channel (800); upper panel). Cell number was determined using WGA-680 (red channel (700); middle panel) and the two channels are merged in the lower panel. **Cii**: Normalized Total ‘In-Cell’ cellular hERG1a expression after a 24 hour pretreatment with phenanthrene (3 or 30 μM) or tamoxifen (20 μM) when compared to control (treatment with P/S and 0.1% DMSO).

Phenanthrene and tamoxifen were applied in DMSO (final concentration of 0.1%). Data are from three independent repeats (n=3). Statistical analysis was performed using One Way ANOVA and a Dunnett’s multiple comparison test; *= *p* <0.05 compared to control (HA-hERG1a + P/S + DMSO).


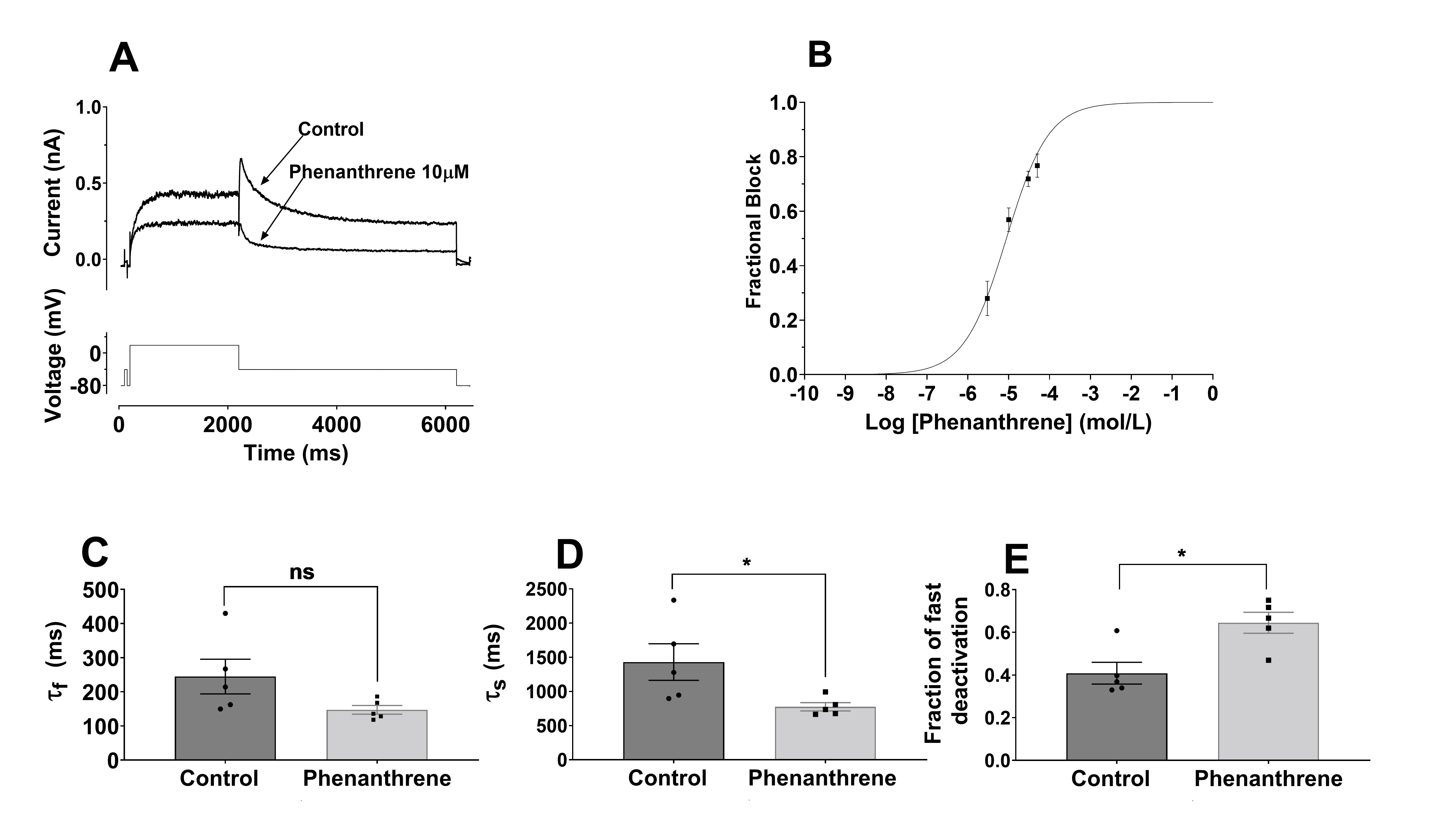


**Supplementary Figure 2: Concentration dependent inhibition of zebrafish ERG (I_zERG_) by phenanthrene.** Phenanthrene inhibited I_zERG_ with a half-maximal inhibitory concentration (IC_50_) of 8.77 ± 0.93 µM, Hill slope: of 0.84 ± 0.09. Phenanthrene also accelerated I_zERG_ deactivation.

**A**: Representative current traces (upper) for Control I_zERG_, and I_zERG_ during application of 10 µM phenanthrene. I_zERG_ was elicited by a voltage protocol (lower) comprised of a 2s depolarizing pulse to +20 mV, followed by repolarization to -40 mV. **B:** Concentration response relation for the effect of phenanthrene on I_zERG_. Four different phenanthrene concentrations between 3 µM and 50 µM were tested: 3 µM, 10 µM, 30 µM and 50 µM. At least 5 replicates were obtained at each concentration.

**C – E:** The bar chart plots compare the deactivation parameters for I_zERG_ in Control (dark grey), and I_zERG_ in the presence of 10 µM phenanthrene (light grey). Biexponential fitting of tail currents on repolarization to -40 mV was used to obtain fast and slow deactivation time-constant values. **C** and **D** show that fast and slow deactivation time constants (τ_f_ and τ_s_ respectively) were reduced by phenanthrene exposure. **E:** compares the proportion of deactivating current described by τ_f_ in control and Phenanthrene. Bar charts show mean ± SEM values, with individual values shown superimposed as filled circles and squares for control and phenanthrene respectively. Significance is denoted by asterisks * *p* < 0.01, (n=5).


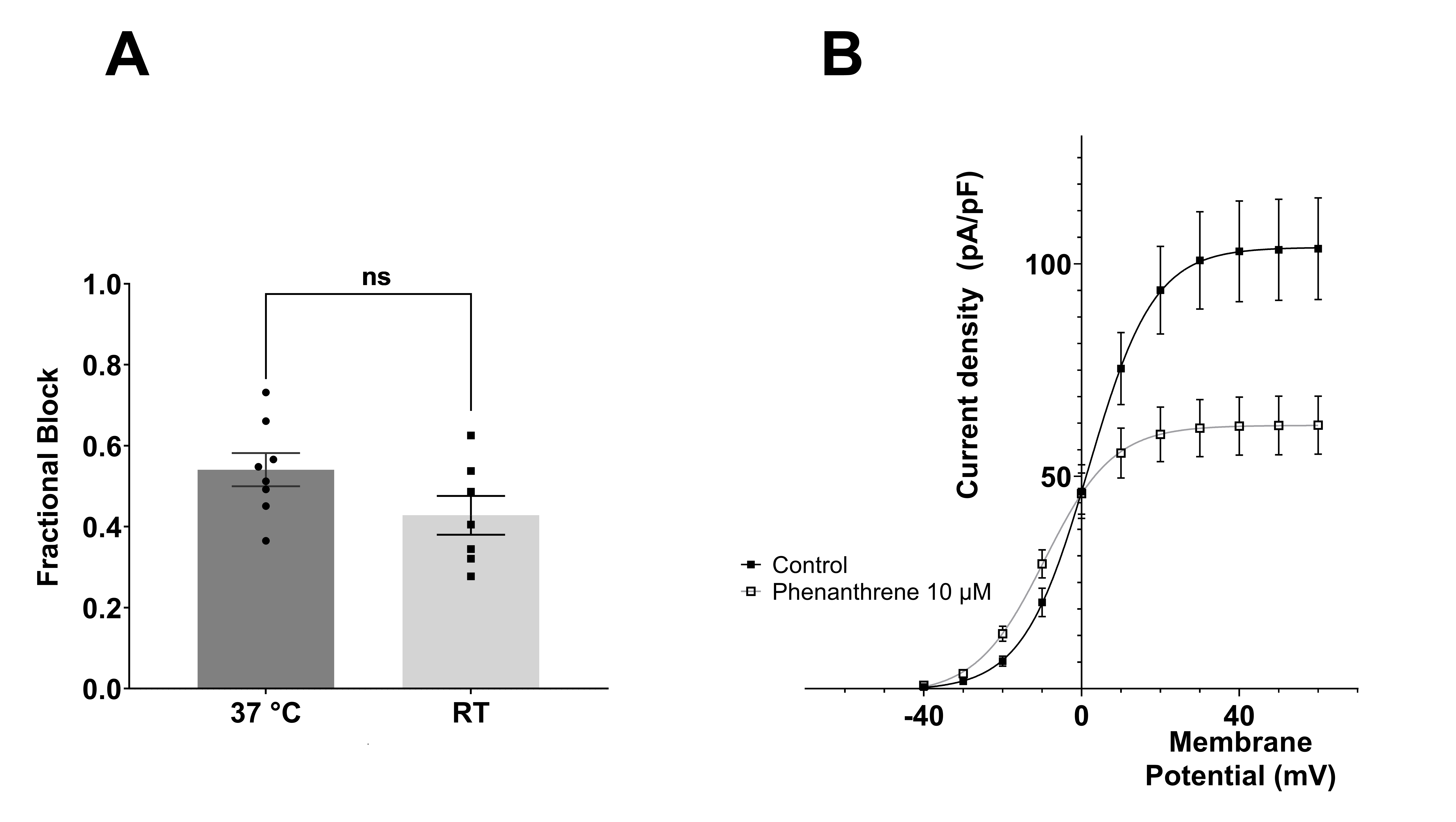


**Supplementary Figure 3: Comparison of phenanthrene inhibition of I_hERG_ at room temperature and 37^o^C and tail current I-V relations.**

**A**: Bar chart showing comparison of mean ± SEM levels of fractional block of I_hERG_ tails at 37^o^C (n=8) and room temperature (RT; n=7) by 10 µM phenanthrene. I_hERG_ from hERG 1a expressing cells was elicited using the standard protocol (+20 mV voltage command, followed by repolarization to -40 mV) used to evaluate concentration-dependent inhibition in main paper Figures 1 and 2 and Supplemental Figure 2. Values from individual experiments are superimposed as filled circles and squares for 37^o^C and RT respectively. There was a tendency towards increased fractional block at the higher temperature, but this did not attain statistical significance (*p*=0.09; unpaired *t*-test).

**B:** Plots of I_hERG_ tail currents (expressed as current density, pA/pF) against test potential for the same experiments in hERG 1a at room temperature shown in Figure 3 of the main paper (n=16). This form of presentation illustrates clearly leftward shifted voltage-dependent activation of I_hERG_ by 10 µM phenanthrene that underpinned the facilitation of current at negative voltages shown in main paper Figure 3. Boltzmann fitting of these mean data yielded control V_0.5_ and *k* values of 1.72 ± 2.45 mV and 8.31 ± 2.21 mV respectively; whilst in phenanthrene the corresponding values were -9.37 ± 3.01 mV and 8.69 ± 2.59 mV respectively. Analysis of means derived from fits to I_tail_ density plots in each condition from individual cells confirmed the leftward shift in activation V_0.5_ was significant (control: 1.39 ± 1.62 mV; phenanthrene -9.47± 1.52 mV; *p* <0.0001 paired *t* test).


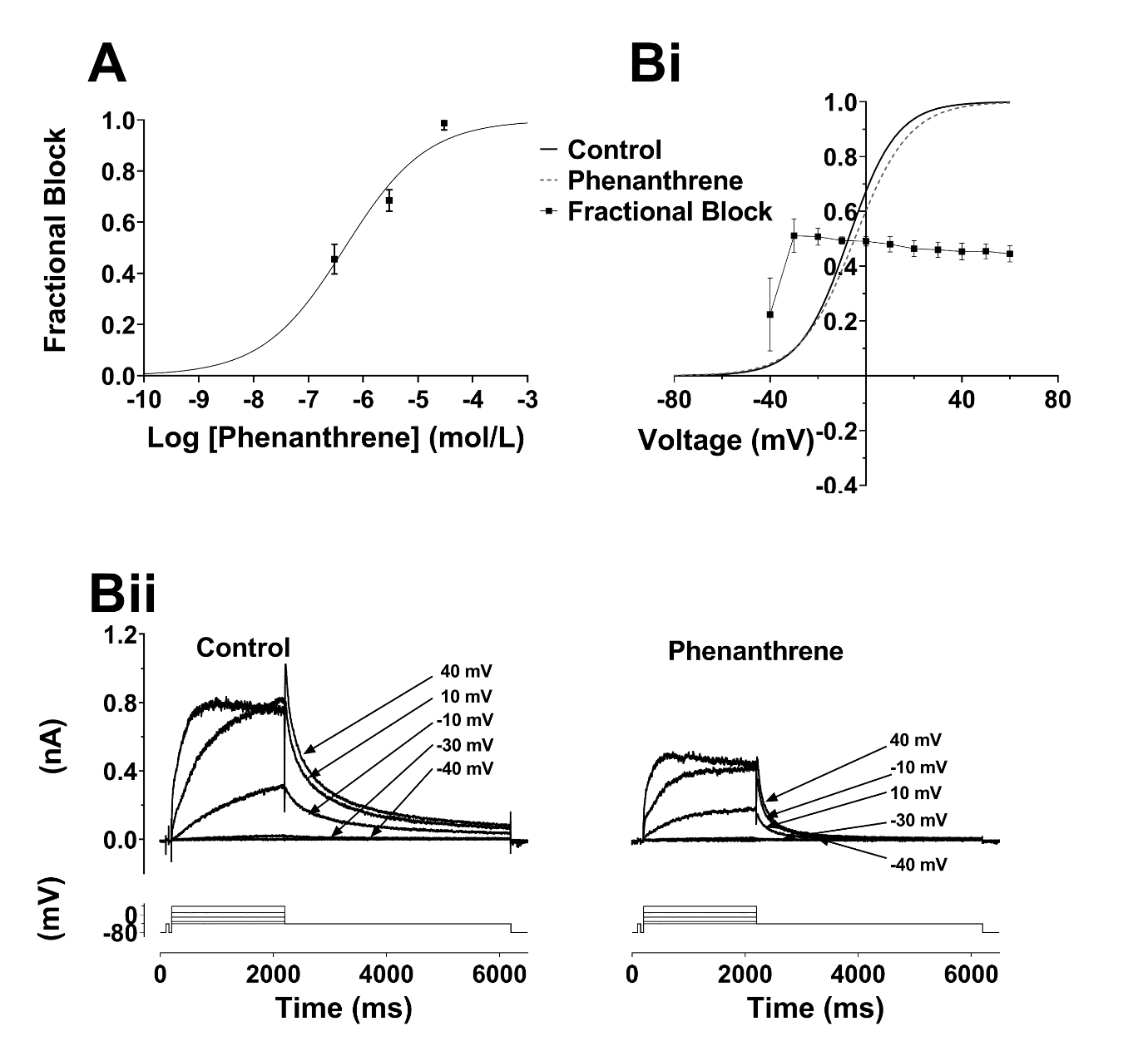


**Supplementary Figure 4: Phenanthrene block of Y652A I_hERG_.** The substitution of the tyrosine residue Y652 with alanine (Y652A) increased the sensitivity of **I_hERG_** to inhibition by phenanthrene. It also largely eliminated the voltage dependence of phenanthrene block seen for wild-type hERG.

**A** Concentration response relation for phenanthrene for Y652A I_hERG_. Three different concentrations between 0.3 µM and 30 µM were tested (0.3 µM n=8, 3 µM n=6, 30 µM n=7). Phenanthrene inhibited Y652A I_hERG_ with a half-maximal inhibitory concentration (IC_50_) of 0.46 ± 0.01 µM, Hill slope: of 0.58 ± 0.11.

**B** Current-voltage (I-V) protocols were applied before and during the application of 0.3 µM phenanthrene. Representative currents are shown in **Bii**. **Bi** Activation relations for I_hERG_ in control and phenanthrene, with superimposed fraction block of I_hERG_ tails following command pulses to each test potential. Activation relations were obtained from mean tail I-V relations in control (black) and 0.3 µM phenanthrene (grey dotted) as described previously (Al Moubarak et al. 2020). Boltzmann fitting to tail I-V relations gave an activation V_0.5_ for Control of -7.3 ± 2.5 mV (n=5; *k*  = 10.09 ± 0.58) and for 0.3 µM of -4.7 ± 1.6 (*p* = 0.06 vs Control, not a significant difference; k = 11.39 ± 0.33, also no significant difference for k, p =0.07), (n=5). This contrasts with the situation for WT I_hERG_ where a significant left-ward shift in activation was seen. Fractional inhibition was flat over virtually the entire voltage range tested. The apparent reduction in fractional inhibition at -40 mV was not statistically significant (One Way ANOVA across the entire voltage range gave a *p* >0.05, F = 2.93).

**Bii** Representative current traces (upper) are shown for Y652A I_hERG_ in Control and Y652A I_hERG_  in 0.3 µM phenanthrene. Voltage protocol (lower) was comprised of 2s command pulses applied at 10 mV increments between −40 mV and +60 mV, followed by repolarization to -40 mV. Only selected traces are shown for clarity of display. Each current trace is labelled with its corresponding test potential.

**Supplementary Figure 5: Effects of phenanthrene on I_hERG_ deactivation for the N588K and S624A hERG mutants.**


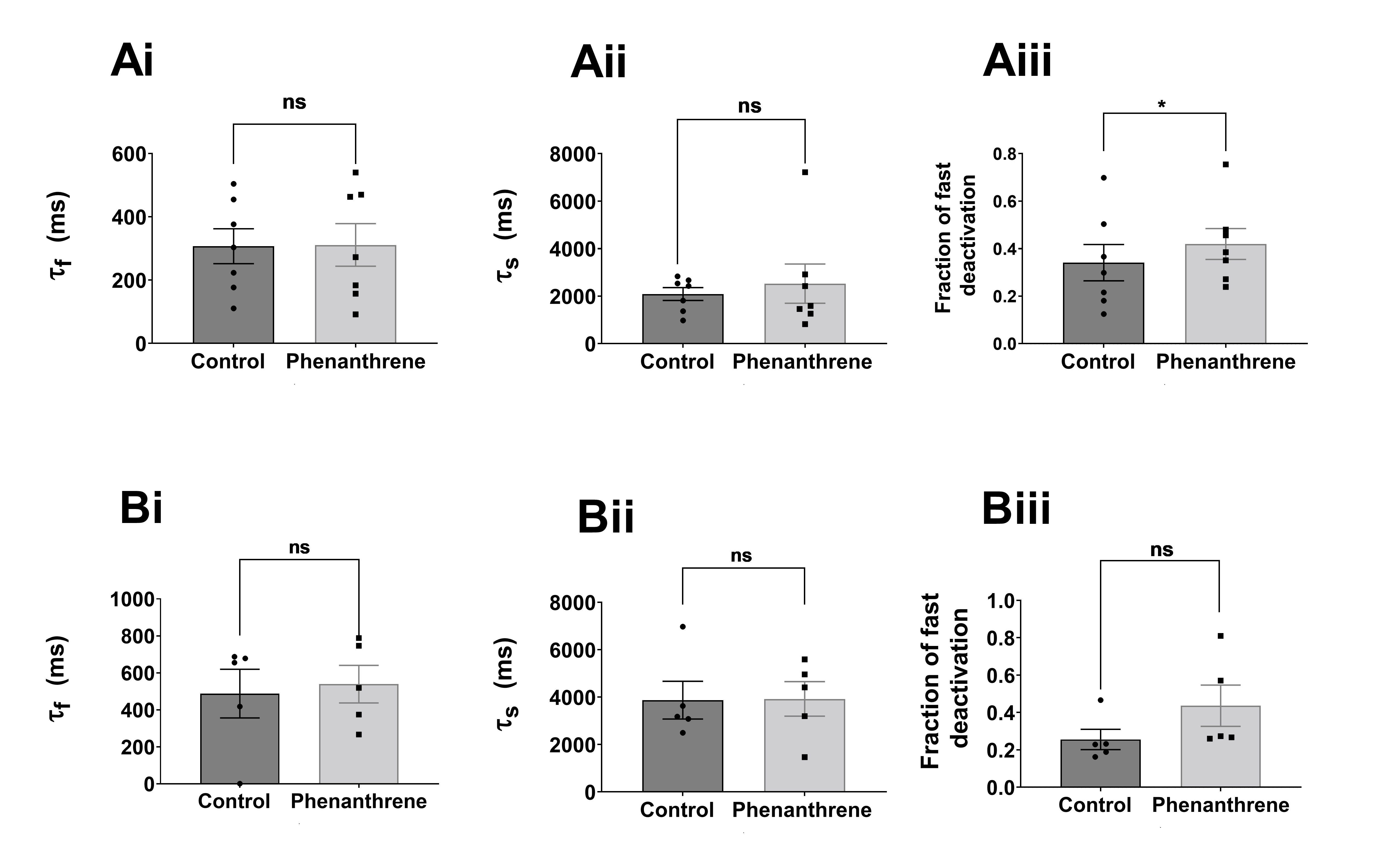


The bar charts compare the deactivation parameters for I_hERG_ in control (dark grey), and 30 µM phenanthrene I_hERG_ (light grey) for N588K hERG and S624A hERG. Tail currents were obtained as shown for Supplementary Figure 2 and biexponential fitting used to obtain fast and slow deactivation time-constants. Bar charts show mean ± SEM values, with individual values shown superimposed as filled circles and squares.

**Ai** and **Aii** show that fast and slow deactivation time constants (τ_f_ and τ_s_ respectively) for N588K hERG. **Aiii:** Compares the proportions of fast deactivating current in control and phenanthrene for N588K hERG. Significance is denoted by asterisks * *p* < 0.05, (n=7). Neither deactivation time constant was altered by phenanthrene, but there was a small increase in the fast component of deactivation.

**Bi** and **Bii** show that fast and slow deactivation time constants (τ_f_ and τ_s_ respectively) for S624A hERG **Biii:** Compares the proportion of fast deactivating current in control and phenenathrene for S624A hERG (n=5). None of the parameters differed significantly between control and phenanthrene for S624A.

For both F557L and M651A mutant channels, I_hERG_ deactivation was sufficiently slow in control solution that it proved difficult to obtain exponential fits to the current tails during the -40 mV repolarisation step. Effects of 30 µM phenanthrene on I_hERG_ deactivation for these mutant channels were therefore quantified by determining the fraction of the peak tail current remaining at the end of the -40 mV repolarisation step. For F557L, in control this fraction was 0.85 ± 0.02 and in phenanthrene it was 0.67 ± 0.04 (*p*<0.01; n=6). For M651A, in control this fraction was 0.72 ± 0.05 and in phenanthrene it was 0.69 ± 0.05 (*p*>0.05; n =5).


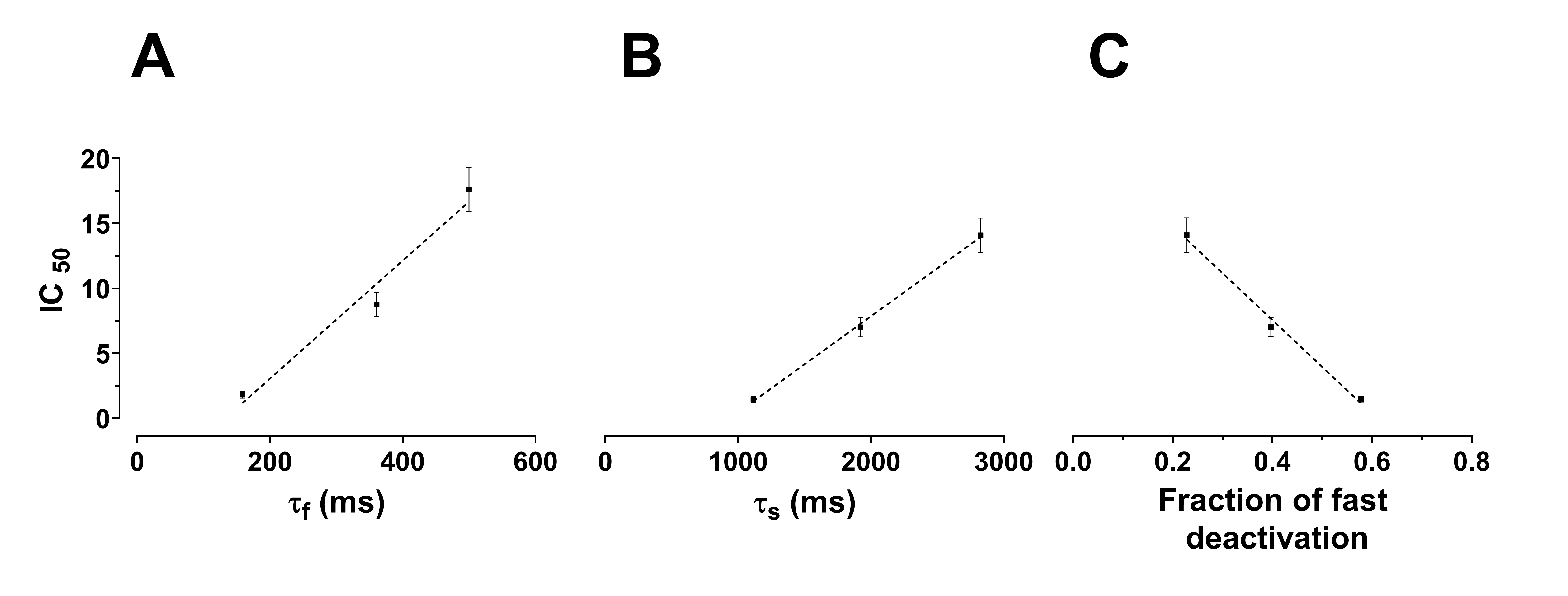


**Supplementary Figure 6: Phenanthrene potency as inhibitor correlates with the deactivation speed in hERG and zERG channels.** The plots show the relationship between the deactivation parameters for 1a I_hERG_ , 1a/1b I_hERG_ , and I_zERG_  in control before phenanthrene application, and corresponding IC_50_ for each of the channels. Simple linear regression was used in the analysis: Slope = (Y_MAX_ -Y _MIN_)/ (X _MAX_ -X_MIN_), Model: Y= Y*Intercept + Slope*X **A** shows IC_50_ values (smallest for I_hERG1a/1b_, intermediate for I_zERG_, largest for I_hERG1a_) plotted against the means of the fast deactivation time constants (τ_f_ ); R = 0.985; R squared = 0.970; Sum of squares: 3.80 **B** shows mean IC_50_ values plotted against means of the slow deactivation time constants (τ_s_) R and R squared = 1, Sum of squares: 0.155

**C:** show IC_50_ values plotted against the proportions of fast deactivating current for each construct; R = 0.996, R squared = 0.992, Sum of squares: 0.974. For all plots the deactivation values plotted are those under control conditions (i.e. prior to phenanthrene application).

**Reference**

1. Al Moubarak E, Zhang Y, Dempsey CE, Zhang H, Harmer SC, Hancox JC (2020) Serine mutation of a conserved threonine in the hERG K^+^ channel S6-pore region leads to loss-of-function through trafficking impairment. Biochem Biophys Res Comm 526: 1085-1091.
